# Revision of the rove beetle genus *Antimerus* (Coleoptera, Staphylinidae, Staphylininae), a puzzling endemic Australian lineage of the tribe Staphylinini

**DOI:** 10.3897/zookeys.67.704

**Published:** 2010-11-10

**Authors:** A. Solodovnikov, A. F. Newton

**Affiliations:** 1 Zoological Museum, Natural History Museum of Denmark, Universitetsparken 15, 2100 Denmark; 2 Field Museum of Natural History, 1400 South Lake Shore Drive, Chicago, Illinois

**Keywords:** Antimerus, new species, eastern Australia, Tasmania, rainforest, larvae

## Abstract

The genus Antimerus Fauvel, 1878, endemic to eastern Australia and Tasmania and a phylogenetically enigmatic member of the large rove beetle tribe Staphylinini, is revised. The genus and each of its four previously known species are redescribed, and a lectotype is designated for Antimerus punctipennis Lea, 1906. Five species are described as new: Antimerus metallicus **sp. n.**, Antimerus jamesrodmani **sp. n.**, Antimerus gracilis **sp. n.**, Antimerus bellus **sp. n.** and Antimerus monteithi **sp. n.**, so that the number of known species in this genus now totals nine. For the first time Antimerus larvae are described, tentatively identified as Antimerus smaragdinus Fauvel, 1878, Antimerus punctipennis and Antimerus metallicus. Available distributional and bionomic data are provided for each species and summarized in the discussion. Adult and larval morphology of Antimerus and its distribution patterns are discussed in the broader context of new data on the evolution of the entire tribe Staphylinini, and with respect to the formation of the Australian fauna of this tribe. The phylogenetic position of Antimerus within Staphylinini remains unresolved pending a targeted formal study. However, a majority of currently available data suggests that it could be a basal member of the recently recovered monophyletic clade of Staphylinini tentatively called “Staphylinini propria”.

## Introduction

In recent years the rove beetle tribe Staphylinini has been a subject of focused morphology-based (Solodovnikov and Newton 2005; Solodovnikov 2006; Solodovnikov and Schomann 2009; Solodovnikov et al., unpublished data) and molecular (Chatzimanolis et al. 2010) phylogenetic studies aimed at producing a consistent, monophyly-based framework for a badly needed new classification of this tribe. Overall, these studies have recovered the following phylogenetic pattern for the tribe: A few “Quediine-looking” genera (e.g., Valdiviodes Smetana, 1981, Astrapaeus Gravenhorst, 1802; all now in the conventional subtribe Quediina) form relictual basal lineages of Staphylinini. Some of these lineages gave rise to the species-rich “northern Quediina” and “southern Quediina” clades. “Northern Quediina” primarily consist of the north temperate species of Quedius Stephens, 1829 (currently a polyphyletic genus, for details see Solodovnikov 2006) and related genera (e.g., Indoquedius Blackwelder, 1952, Quetarsius Smetana, 1996). “Southern Quediina” is a lineage consisting of the south temperate species of Quedius (many of which occur in Australia), some south temperate genera of Quediina (e.g., Loncovilius Germain, 1903, Cheilocolpus Solier, 1849, Quediomimus Cameron, 1948), global Heterothops Stephens, 1829 (all currently in Quediina) and Atanygnathus Jakobson, 1909 (member of the currently monotypic subtribe Tanygnathinina), as well as the current subtribe Amblyopinina. Another species-rich and robust clade tentatively called “Staphylinini propria” (Chatzimanolis et al. 2010) includes the current subtribes Staphylinina, Philonthina, Xanthopygina and Anisolinina. With only minor exceptions, the current subtribes Staphylinina and Philonthina seem to be largely monophyletic. Monophyly of the current subtribes Xanthopygina and Anisolinina, on the contrary, was questioned. Only the Neotropical core of Xanthopygina seems preliminarily to form a monophyletic group, and the composition and affinities of Anisolinina seem to be even more doubtful. With the introduction of these new phylogenetic data, sister-group relationships of several genera of Staphylinini which were traditionally either *incertae sedis* within Staphylinini, or only tentatively assigned to one of its formal subtribes, have been clarified.

However, there are still a number of genera of Staphylinini for which phylogenetic affinities within the tribe are completely unclear, and the genus Antimerus, the subject of this paper,is one of them. It was originally based on a single new species, Antimerus smaragdinus Fauvel, 1878, from southeastern Australia (Fig. 11), and placed in the staphylinine group Quediini close to the Japanese genus Algon Sharp, 1874 (Fauvel 1878). A further three species from eastern Australia were added by (Lea (1906, 1925), two of them with doubts about their generic assignment, but without further comment on the placement of the genus. The genus remained in the tribe Quediini or subtribe Quediina (of Staphylinini) in the Coleopterorum Catalogus (Bernhauer and Schubert 1916: 410; Scheerpeltz 1933: 1420) and in Blackwelder (1952: 57). In the most recent printed world catalog of Staphylinidae (Herman 2001: 165, 3571), the genus appears instead in the subtribe Xanthopygina, without comment. This new subtribal assignment for Antimerus was consistent with redefinitions of Quediina, Xanthopygina and some other subtribes of Staphylinini by (Smetana (1977, 1985) and Smetana and Davies (2000), although Antimerus was not specifically mentioned in those works. In the Field Museum-based on-line database of the genus-group names of Staphylinoidea (Newton and Thayer 2005a) Antimerus, instead, is listed as Staphylinini *incertae sedis*.

Of all these recent papers cited above, Antimerus was included in a formal analysis only in Solodovnikov (2006), where its position was resolved with a high degree of ambiguity. That analysis implies two alternative hypotheses: either Antimerus is a very basal and ancient lineage of Staphylinini, similar to an enigmatic Australian endemic Lonia Strand, 1943 (Fig. 6a in Solodovnikov 2006); or, like a puzzling genus Algon, it is an isolated, presumably basal, member of “Staphylinini propria”, however not fitting any of its formal subtribes (Fig. 6b in Solodovnikov 2006). Nevertheless, that and other exploratory analyses (Solodovnikov, unpublished) clearly indicate that Antimerus does not belong either to “northern Quediina” where the type species of the formal subtribe Quediina belongs, or to “southern Quediina” that constitute the largest part of the Australian fauna of Staphylinini. Also, it does not seem to be linked to the core (Neotropical species only) of the subtribe Xanthopygina.

At the local (Australian) scale, the biogeographic history of such odd genera as Antimerus (as well as some others, for example Lonia Strand, 1943 (Fig. 20) or Australotarsius Solodovnikov & Newton, 2009) is of high interest. Although there have been no rigorous attempts to time-calibrate the discussed newly emerging phylogenetic pattern of Staphylinini, the hitherto available phylogenetic (listed above) and paleontological data on Staphylinidae (Solodovnikov, unpublished) make it plausible to date the origin of Staphylinini back to Late Jurassic or Early Cretaceous, and to associate divergence between “northern Quediina” and “southern Quediina” with separation of Gondwana and Laurasia. Considering the recent distribution pattern of the group, it also seems plausible to assume that the origin and initial diversification of the younger “Staphylinini propria” clade took place in the northern hemisphere landmasses, with subsequent dispersal of some of its lineages southwards. Within such a framework, it appears quite evident that the Australian fauna of Staphylinini (for a complete catalogue of taxa see Newton and Thayer 2005b) predominantly consists of species belonging to the “southern Quediina” lineage, which have been evolving in this continent *in situ* for a long time (meaning the “continent” in a broader Gondwanan sense than modern Australia only). And it is also evident that the Australian fauna is very depauperate as far as the “northern Quediina” and “Staphylinini propria” are concerned. Only a small fraction of the genera and species of these alien lineages were able to disperse to Australia, either naturally or via human-induced introduction. Whether Antimerus is an ancient relict in Australia, or a younger arrival to this continent from the north, remains to be understood.

As a step towards a better overall knowledge of Antimerus, this really peculiar element of the Australian fauna of Staphylinini, we provide here its taxonomic revision, including description of five new species (out of nine species in total) and description of its presumed larva.

## Materials and methods

This paper is based on the study of specimens using high quality dissecting microscopes. Material is kept in several institutions, listed below (with names of responsible collection personnel) and cited in the “Material examined” sections by the indicated coden or name. Beetles were examined mainly as pinned dry specimens, but a few were macerated in 10% KOH, rinsed, disarticulated and examined as wet preparations in glycerin to produce a more detailed generic redescription. The same wet procedure was applied for a study of alcohol-preserved larvae, and for beetle aedeagi. All line illustrations were made using a camera lucida. All measurements are given in millimeters; they were made with an ocular linear micrometer and abbreviated as explained below. All specimens were databased at the Field Museum, and most were assigned a unique “FMNH-INS” number (if no existing unique number was present). These numbers are cited in the “Material examined” sections only for holotypes, lectotype, and paratypes of rare species. Specimen labels for primary types (holotypes, lectotype) are cited exactly in quotes, with a slash (/) separating lines; data for other specimens are generally cited as given on labels, except that collecting dates are standardized in the formula day.month.year, using lower-case Roman numerals for month. Data not present on original labels but added for clarity or amplification (e.g., coordinates) are given in square brackets [ ].

### Collections and their abbreviations

AMSAustralian Museum, Sydney (D. Britton)

ANICAustralian National Insect Collection, Canberra (A. Ślipiński, T. Weir, C. Lemann)

BMNHThe Natural History Museum, London (R. Booth)

BPBMBernice P. Bishop Museum, Honolulu (S. Myers, A. Samuelson)

CASCalifornia Academy of Sciences, San Francisco (D. Kavanaugh)

FMNHField Museum of Natural History, Chicago (M. Thayer, J. Boone)

Lorimerprivate collection of Vincent Lorimer, Australia

MCZMuseum of Comparative Zoology, Harvard University, Cambridge (P. Perkins)

MVMAMuseum of Victoria, Abbotsford (C. McPhee, A. Neboiss)

NHMWNaturhistorisches Museum, Wien (H. Schillhammer)

Porchprivate collection of Nicholas Porch, Australia

QDPCQueensland Department of Primary Industries, Indooroopilly (J. Donaldson)

QMQueensland Museum, South Brisbane (G. Monteith, G. Thompson)

QPIMQueensland Department of Primary Industries, Mareeba (R. Storey)

SAMSouth Australian Museum, Adelaide (J. Forrest)

TMSATransvaal Museum, Pretoria (J. Harrison, R. Müller)

UCDCR.M. Bohart Museum of Entomology, University of California, Davis (S. Heydon)

UQICUniversity of Queensland, St. Lucia (G. Daniels)

VAICVictorian Agricultural Insect Collection, Knoxfield (K.L. Dunn)

ZMHBMuseum für Naturkunde der Humboldt Universität, Berlin (M. Uhlig)

ZMUCZoological Museum, University of Copenhagen, Copenhagen (O. Martin)

ZMUNZoological Museum, University of Oslo, Oslo (V. Gusarov)

### Measurements and their abbreviations

HL– head length (from apex of clypeus to neck constriction); HW– head width (maximal, including eyes); PL– pronotum length (along median line); PW– pronotum width (maximal); EL– elytral length (from humerus to most distal apical margin; best taken from lateral view of the elytron); EW– combined width of both elytra (maximal, elytra closed along suture). The total length of the body given in the descriptions was measured from the tip of the labrum to the tip of the abdomen.

## Results

### Genus Antimerus

Fauvel, 1878

Antimerus Fauvel 1878: 550.

#### Type species:

Antimerus smaragdinus Fauvel, 1878 (by monotypy).

#### Diagnostic description.

(Figs 1–18, 21–42). Large Staphylinini (13–20 mm extended), robust, more or less parallel-sided with head about as wide as pronotum, elytra and abdomen (Figs 9–18).

**Figures 1–8.**
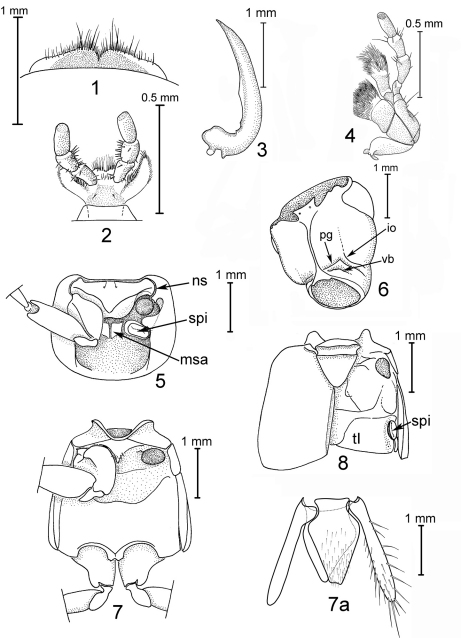
Morphology of the genus Antimerus (based on Antimerus smaragdinus): **1** labrum dorsally **2** labium ventrally **3** right mandible dorsally) **4** left maxilla ventrally **5** prothorax ventrally (left anterior leg removed) **6** head capsule latero-ventrally **7** meso- and metathorax, ventrally (left middle leg removed) **7a** male terminalia ventrally (sternite IX, tergum X and lateral sclerites of tergite IX, chaetotaxy shown on sternite IX and left lateral sclerite only) **8** meso- and metathorax with fused abdominal tergite I, dorsally (right elytron removed); *io*, infraorbital ridge, *msa*, median sclerotized area; *ns*, notosternal suture; *pg,* postgenal ridge; *vb,* ventral basal ridge; *spi*, spiracle; *tI*, abdominal tergite I.

Head transverse; neck about half as wide as head, distinct at sides but not or indistinctly marked dorsally; eye large, occupying more than half of side of head; temple short, hind angle of head broadly rounded or indistinct; antenna slender, longer than head width, increasingly densely pubescent from about antennomere 6 to apex; labrum (Fig. 1) very short and wide, about 2/3 as wide as head, with sclerotized anterior margin and acute median emargination; mandible (Fig. 3) long, slender, falcate, about as long as head excluding neck, with narrow bladelike medial edge along apical half or more, edentate or at most with one small tooth along medial edge of basal half, with short abruptly expanded base, and without prostheca (except Antimerus auricomus with very slender prostheca no longer than width of mandible at base); maxillary (Fig. 4) and labial (Fig. 2) palps with robust, elongate apical palpomeres that are about as wide or wider than more basal palpomeres and with abruptly truncate apices; galea and lacinia densely setose; paraglossa very long, fingerlike, with comb of strong setae along mesial edge; glossae short, densely setose; prementum small, transverse; mentum transverse, broadly emarginate at apex; gular sutures (Fig. 6) complete, closely approximate through much of length; postgenal (Fig. 6, pg) and ventral basal (Fig. 6, vb) ridges well-developed, nuchal ridge absent, infraorbital ridge (Fig. 6, io) rudimental, short.

**Figures 9–14.**
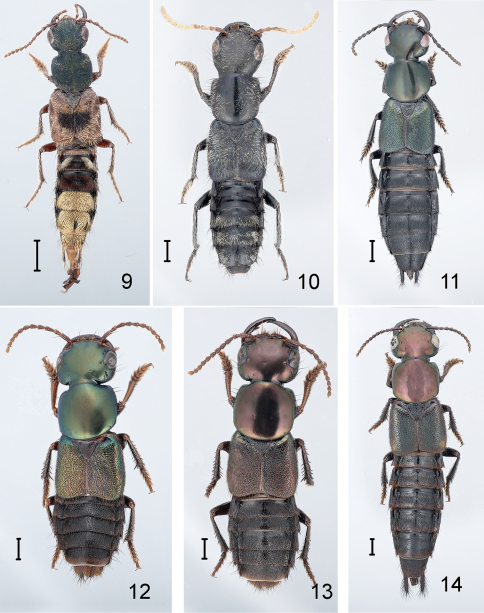
Species of Antimerus, habitus: **9** Antimerus auricomus **10** Antimerus posttibialis **11** Antimerus smaragdinus **12, 13** Antimerus punctipennis **14** Antimerus metallicus. Scale bars equal 1 mm.

Pronotum (Fig. 5) subquadrate to strongly transverse; superior line of hypomeron continued onto anterior margin, visible from above (except in Antimerus posttibialis where it is deflexed ventrad and not visible from above in apical fourth); inferior line of hypomeron complete except interrupted at coxal articulation, or more or less obsolete; hypomeron with large triangular partly translucent post-coxal process; hypomeron completely visible from side (except in Antimerus monteithi sp. n. where it is inflexed and completely hidden in lateral view except for apex of postcoxal process); notosternal suture (Fig. 5, ns) complete, distinct; prosternum short, transverse, its anterior edge laterally not forming an abrupt angle with hypomeron; procoxal cavities largely closed behind by very large mesothoracic spiracles (Fig. 5, spi) plus a smaller irregular median sclerotized area (Fig. 5, msa) between the spiracles.

Scutellum large, triangular, with two transverse subbasal carinae. Elytron without epipleural ridge; elytra together about as long as wide. Hind wings fully developed and functional, each with completely separate MP4 and CuA veins. Mesocoxae contiguous, mesocoxal cavities delimited anteriorly and posteriorly by carinae (Fig. 7). All tarsi 5-segmented, tarsomeres 1–4 of all legs of both sexes more or less broad, basal tarsomeres of front legs as wide as or wider than width of protibia, those of other segments narrower than width of corresponding tibiae; tarsomeres 1–4 of all legs with dense brushes of tenent setae ventrally; tarsal claws simple, empodium with pair of setae that are shorter than claws.

Abdomen without prototergal gland (Fig. 8), apparently without eversible defensive glands posterior to tergum VIII. Tergites II–VIII and sternites III–VIII each with a single subbasal carina that is curved posteriad at sides (behind spiracle on tergites); tergites III–VIII also with short oblique anterolateral carina before spiracle. Segments III–VII with two pairs of laterotergites each, the dorsal laterotergites on III–VI each with a subbasal carina. Intersegmental membranes between segments III–VII attached preapically to preceding segment, with irregular or quadrangular, more or less rounded sclerites occupying less than 70% of membrane surface. Sternum VIII with slight to moderate apical emargination in male, with no or more shallow emargination in female; sexual dimorphism otherwise restricted to genital segment and associated genitalia.

Genital segment of both sexes with triangular tergum X which widely separates lateral tergites IX dorsally, and each lateral tergite IX produced into hollow, fingerlike, apically obtuse or rounded process. Male sternite IX entire, more or less symmetrical or with base slightly more produced anteriad on left side (Fig. 7a). Aedeagus (Figs 21–42) with parameres fused into a single large apically emarginate lobe which bears dense field of dark peg setae facing median lobe, and at least 4 pairs of apical setae; median lobe elongate, tubular for most of length with basal membranous area bearing pair of flaps, apex more or less triangularly produced to extend slightly beyond apex of paramere; internal sac with large well sclerotized copulatory sclerite that abuts apex of median lobe when internal sac everted, and pair of more distal membranous or partly sclerotized lobes; aedeagus in repose in abdomen with paramere facing left side of beetle. Female without median ventral sclerite, ovipositor consisting of paired proximal and distal gonocoxites, styli apparently absent; spermatheca apparently not sclerotized.

#### Distribution and bionomics.

(Figs 55, 56) All hitherto known species of Antimerus (see below) are confined to the moist forests of the coastal hills and mountain ranges of eastern Australia, from northern Queensland to Victoria, South Australia and Tasmania. However, there is a significant gap in this arc: the genus is not known to occur for a long stretch in central and southern Queensland (Fig. 55, A–C). This seems to be not a sampling artifact, but a real disjunction coinciding with the gap in the distribution of moist forests in eastern Australia (Groves 1994). Apart from evidence that species of Antimerus are apparently confined to forests (e.g., Fig. 56), very little is known about their microhabitat preferences, which seem to vary from one species to another (see species details below). Available label data, our own collecting experience, and morphology of the genus (large eyes, expanded tarsi of all legs) suggest that at least some species of the genus are diurnal and more or less arboreal. Arboreality may explain their rarity in collections, which have been mostly obtained by methods targeting microhabitats on or near the ground.

#### Comparison.

In Australia, Antimerus can be distinguished from any other genus of the tribe Staphylinini by the following combination of characters: relatively large body size (13–20 mm); relatively long falcate mandibles without distinct teeth internally (except small tooth on left mandible only in two species); deflexed hypomera of pronotum visible in lateral view (except Antimerus monteithi, concealed); tarsomeres 1–4 of all legs in both sexes broad and bearing tenent setae ventrally; and one pair of empodial setae on all tarsi. The only sympatric genus likely to be confused with Antimerus is Lonia which has distinct mandibular teeth internally and simple meso- and metatarsi (Fig. 20).

**Figures 15–20.**
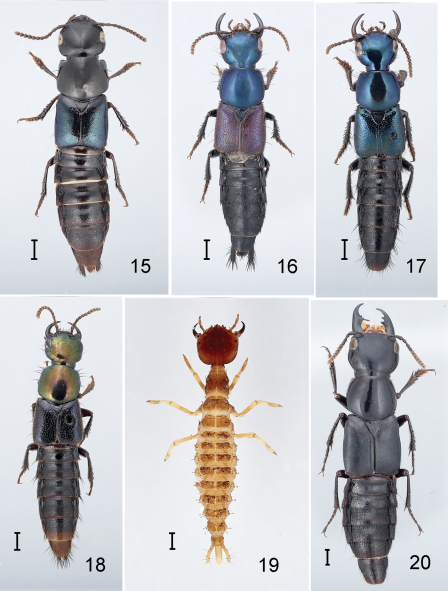
Species of Antimerus, adults and larva, and Lonia regalis: **15–18** Antimerus, adults: **15** Antimerus monteithi **16** Antimerus jamesrodmani **17** Antimerus bellus **18** Antimerus gracilis **19** Antimerus punctipennis, presumed larva (instar III) **20** Lonia regalis. Scale bars equal 1 mm.

#### Key to Antimerus species (adults)

**Table:** 

1.	Disc of head and pronotum moderately densely punctate and setose; body vestiture including pale (white or yellow) as well as dark setae, with dense patches of pale setae on at least some abdominal terga (Figs 9, 10); left mandible with small but distinct tooth at middle of medial edge	2
–	Disc of head and pronotum impunctate, glabrous; body vestiture entirely dark, without dense patches of setae on abdominal terga (Figs 11–18); mandibles edentate or with minute tooth in basal third	3
2(1).	Body multicolored: head and pronotum with blue-green reflection, elytra red with black medial spot, abdomen dark with terga VI–VIII covered with dense yellow pubescence; head and pronotum with coarse “fingerprint whorl” microsculpture, dull (southern Queensland, New South Wales)	Antimerus auricomus (Figs 9, 21–23)
–	Body entirely dark brown to black, abdominal terga V–VII with dense pale pubescence on apical half; head and pronotum with very fine microsculpture of mostly parallel transverse lines, weakly shining (northern Queensland)	Antimerus posttibialis (Figs 10, 24–26)
3(1).	Head and pronotum without metallic reflection; pronotum strongly transverse, ca. 1/5 wider than long; hypomeron inflexed and not visible from side except for apex of postcoxal process; macrosetae absent from anterior medial edge of eye and anterior margin of pronotum; disc of elytron glabrous (southern Queensland, northern New South Wales)	Antimerus monteithi sp. n. (Figs 15, 40–42)
–	Head and pronotum with metallic bronze, green or blue reflection; pronotum less transverse to quadrate, not more than 1/10 wider than long (Figs 11–14, 16–18); hypomeron completely visible from side; 1 or more macrosetae present at anterior medial edge of eye and along anterior margin of pronotum; disc of elytron moderately densely punctate and setose	4
4(3).	Disc of head and pronotum with very fine dense reticulate microsculpture, rather dull; elytral surface between punctures rugose or pitted, thus also dull (Figs 11–13)	5
–	Disc of head and pronotum with very fine microsculpture of mostly parallel transverse lines, moderately shining; elytral surface between punctures more or less smooth, moderately shining (Figs 14, 16–18)	
65(4).	Head, pronotum and elytra generally with metallic bronze reflection, rarely greenish; elytral punctures larger and more dense, separated on average by about the diameter of one puncture, the surface between punctures irregularly rugose but not pitted; abdominal terga evenly transversely convex (southern Queensland, New South Wales)	Antimerus punctipennis (Figs 12, 13, 30–33)
–	Head, pronotum and elytra generally with metallic green or blue reflection; elytral punctures smaller and less dense, separated on average by about twice the diameter of one puncture, the surface between punctures very finely pitted; abdominal terga with vague elevation along midline (southern New South Wales, Victoria, South Australia, Tasmania)	Antimerus smaragdinus (Figs 11, 27–29)
6(4).	Larger, length extended ca. 16–20 mm; antennomeres 8–10 about as long as wide; head and pronotum generally with metallic bronze reflection, rarely greenish; elytral punctures larger and more dense, separated on average by less than twice the diameter of a puncture; abdominal terga usually with vague elevation along midline (northern Queensland)	Antimerus metallicus sp. n. (Figs 14, 30–32, 34)
–	Smaller, length extended ca. 13–15 mm; antennomeres 8–10 strongly transverse; head and pronotum with metallic blue or green reflection (Figs 16–18); elytral punctures smaller and less dense, separated on average by more than twice the diameter of a puncture; abdominal terga evenly transversely convex	7
7(6).	Neck delimited from head dorsally by very fine groove; elytra red (southern Queensland)	Antimerus jamesrodmani sp. n. (Figs 16, 35–37)
–	Neck not at all delimited from head dorsally; elytra dark, with or without metallic reflection (Figs 17, 18)	8
8(7).	Head only slightly narrowed behind eyes, with distinct temples; head and pronotum with bright metallic green reflection, elytra with only vague metallic bronze reflection; abdomen dark with apical third of segment VII and all of VIII and genital segment red (northern Queensland)	Antimerus gracilis sp. n. (Fig. 18)
–	Head rapidly narrowed behind eyes, without distinct temples; head, pronotum and elytra with similar metallic blue-green reflection; abdomen entirely dark (New South Wales)	Antimerus bellus sp. n. (Figs 17, 38, 39)

### Antimerus auricomus

Lea 1925

Figs 9 21–23

Antimerus auricomus Lea 1925: 229

#### Type locality:

AUSTRALIA: New South Wales: Dorrigo [30°20’S, 152°50’E]

#### Material examined.

**AUSTRALIA: New South Wales:** Holotype, mounted on card, genitalia not dissected, with labels:“auricomus / Dorrigo / Lea, TYPE”, “20359 / Antimerus / auricomus Lea / N. S. Wales / TYPE”, “S. Aust. Museum / specimen” [orange label], “FMNH-INS 0000 019 152”, “HOLOTYPE / Antimerus / auricomus Lea / revised by / A. Solodovnikov 2006” [red label], ♂ in SAM. Other material: Bagawa State Forest, 1260 Road, 9 km SW by S of Glenreagh, 30°7’S, 152°50’E, 17.xi.1983 (D.C.F. Rentz & M.S. Harvey), 1♂ in ANIC; Barrington Tops, 5000 ft [1525m], 13.i.1947 (L. Hopson, A. Musgrave), 1♂ in AMS; Barrington Tops, Big Hole, 21.i.1979, on trunk of Eucalyptus sp. (G.R. Brown), 1♀ in ANIC; Macksville, xii.1990 (F. Wachtel), 1♂ in NHMW; **Queensland:** Mt. Glorious S.F. (NW Brisbane), 750m, 27°23’S, 152°45’E, subtropical rainforest, 17–24.xii.1987, ex canopy (25m) Agyro. actinoph., IT2 (0FT) (Y. Basset), 1♂ in ZMUC; 24–31.xii.1987, ex canopy (25m) Agyro. actinoph., IT2 (0FT) (Y. Basset), 1♂ in FMNH; 7–14.i.1988, ex canopy (25m) Agyro. actinoph., IT2 (0FT) (Y. Basset), 1♂ in FMNH; 5–12.ii.1987, ex canopy (25m) Agyro. actinoph., IT4 (0FT) (Y. Basset), 1♂ in FMNH; 24–31.xii.1987, ex canopy (25m) Agyro. actinoph., IT4 (0FT) (Y. Basset), 1♀ in FMNH; Nat. Pk., 22.ii.1933, 1♀ in QM.

#### Description.

Measurements (n=5): HL: 1.7–2.4; HW: 2.4–2.9; PL: 2.3–2.7; PW: 2.4–3.1; EL: 3.6–4.0; EW: 2.7–3.7. Total length of the body 14–16 mm.

Head and pronotum metallic blue with purple reflection, with dense microsculpture resembling “fingerprint whorl”, with moderately dense punctuation and pale pubescence; elytra brown with dark spot in the middle of disc, with dense punctuation and dense pubescence; abdomen dark brown to black, third and fourth tergites (first and second visible) with patches of dense silver pubescence laterally, fifth tergite (third visible) with black pubescence, sixth to eight tergites (fourth to sixth visible) with dense yellow pubescence; appendages brown. Body elongate, slender.

Head slightly wider than long, with tempora rapidly narrowing towards neck so that posterior angles of head indistinct; tempora as long as eye (in lateral view). Left mandible with very small but distinct tooth in the middle of its internal edge; right mandible with even smaller tooth in the same position. Antennae moderately long, with antennomeres VIII–X about as long as wide, not transverse.

Pronotum about as long as wide and as wide as head, widest before (anterior) to its middle; pronotal anterior angles distinct, posterior broadly rounded; hypomera not inflexed, visible from lateral view.

Elytron elongate, considerably longer than pronotum; elytral surface between punctures with distinct microsculpture consisting of isodiametric cells, dull.

Wings well developed.

Abdominal tergites III–VI (first to fourth visible) with deep transverse impression in basal part; tergite VII (fifth visible) with whitish seam at apical margin.

**Figures 21–26.**
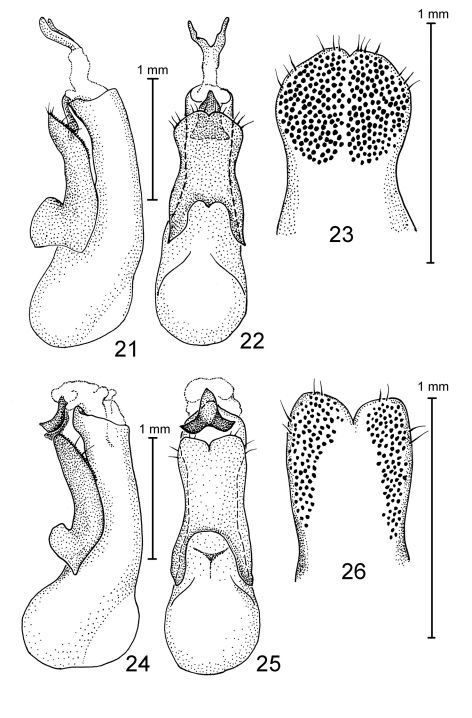
Species of Antimerus, aedeagi: **21–23** *auricomus* **24–26** *posttibialis* **21, 24** aedeagus laterally **22, 25** aedeagus dorsally (parameral side) **23, 26** apical portion of paramere, underside.

Male (Figs 21–23). Aedeagus with relatively wide (wider than median lobe in dorsal or ventral view) paramere, which is slightly notched at the apex. Sclerotized piece of internal sac triangle-shaped.

#### Comparison.

Among all other species of the genus, Antimerus auricomus can be easily recognized by the very characteristic coloration of the body: metallic blue with purple forebody, pale brown elytra with darker spot, and black abdomen with multicolored pubescence.

#### Distribution and bionomics.

Antimerus auricomus is known from several localities in eastern Australia, in southern Queensland and northern New South Wales (Fig. 55, C, circles). Specimens with more complete label data were collected in subtropical rainforest from the canopy of the tree Argyrodendron actinophyllum Edlin (Sterculiaceae) 25 m above ground (see Basset 1991), and on the trunk of a Eucalyptus tree.

#### Notes on type material.

There is a clear indication in the original description that the latter was based on a single specimen (“New South Wales: Dorrigo (unique))”, which was unambiguously located at SAM and interpreted as holotype.

### Antimerus posttibialis

Lea 1925

Figs 10 24–26

Antimerus posttibialis Lea 1925: 229

#### Type locality:

Australia: Queensland:Kuranda [16°49’S, 145°39’E]

#### Material examined.

**AUSTRALIA: Queensland:** Holotype, pinned, genitalia not dissected, with labels:“Lea, TYPE / posttibialis / Kuranda”, “12098 / Antimerus / posttibialis / Lea / Queensland / TYPE”, “S. Aust. Museum / specimen” [orange label], “FMNH-INS 0000 019 154”, “HOLOTYPE / Antimerus / posttibialis Lea / revised by / A. Solodovnikov 2006” [red label], ♂ in SAM. Other material: same locality, ii.1921 (F.P. Dodd), 1♂ in SAM; same locality [no date] (F.P. Dodd), 1♂ in SAM; same locality, ii.1909 (G.E. Bryant), 1♀ in ANIC, 2♂ in BMNH; same locality, 1911 (G.E. Bryant, F.P. Dodd), 1♂ in FMNH; NEQ: Mt. Murray Prior, 770m, 16°56’S, 145°51’E, 31.x.1995, pyrethrum, trees & rocks (Monteith & Cook), 1♀ in QM.

#### Description.

Measurements (n=5): HL: 2.5–2.9; HW: 3.1–3.5; PL: 2.9–3.2; PW: 2.8–3.5; EL: 3.5–4.2; EW: 3.5–4.2. Total length of the body 16–17 mm.

Body black, without distinct metallic reflection; elytra near their apical margin, and legs slightly paler; two to three basal segments of antennae pale brown, rest of antennae yellowish. Head, pronotum and elytra with moderately dense punctuation and pale, yellowish pubescence, their surfaces at interspaces with microsculpture of mostly transverse waves. Fifth to eighth abdominal tergites (third to sixth visible) with patches of more or less dense silver pubescence. Body elongate, relatively slender.

Head slightly wider than long, with tempora gradually tapering to weakly defined neck constriction, so that posterior angles of head indistinct; tempora as long as eye (in lateral view). Left mandible with very small but distinct tooth in the middle of its internal edge; right mandible without such tooth. Antennae moderately long, with antennomeres VIII–X about as long as wide, not transverse.

Pronotum about as long as wide and as wide as head, its lateral sides gradually diverging from base to pronotal anterior margin, and slightly converging very near to anterior angles; anterior angles very distinct, posterior slightly distinct. Pronotal hypomera not inflexed, visible from lateral view.

Elytron elongate, distinctly longer than pronotum.

Wings well developed.

Abdominal tergites III–VI (first to fourth visible) with deep transverse impression in basal part; tergite VII (fifth visible) with whitish seam at apical margin.

Male (Figs 24–26). Aedeagus with paramere as wide as median lobe (in dorsal or ventral view); paramere strongly notched at the apex. Sclerotized piece of internal sac with shape of three-lobed structure.

**Figures 27–34.**
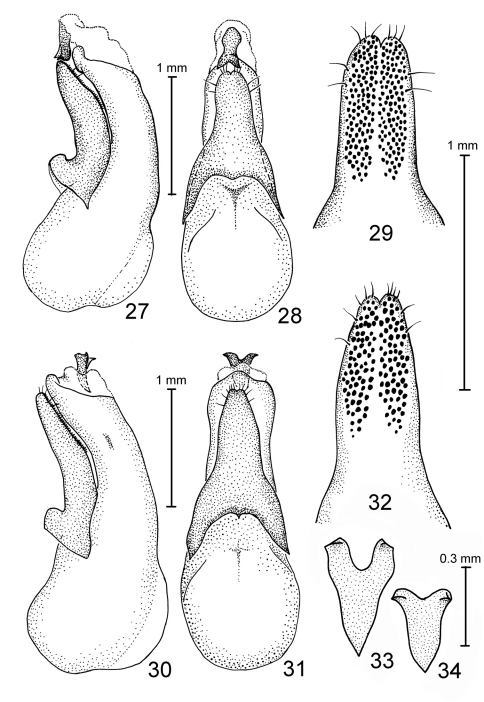
Species of Antimerus, aedeagi: **27–29** *smaragdinus* **30–33** *punctipennis* **34** *metallicus* **27, 30** aedeagus laterally **28, 31** aedeagus dorsally (parameral side) **29, 32** apical portion of paramere, underside **33, 34** sclerite of the internal sac.

#### Comparison.

From all other species of the genus, Antimerus posttibialis can be easily recognized by the combination of the black, non-metallic coloration of the body covered by silver pubescence, and patches of dense silver hairs on the abdominal tergites V–VIII (third to sixth visible).

#### Distribution and bionomics.

Antimerus posttibialis is known from only a few specimens from two localities in northern Queensland, near the eastern coast of Australia (Fig. 55, C, triangles). Habitat data are recorded for only one specimen, which was collected in the rainforest at 770 m elevation by low scale pyrethrum fogging of tree trunks and rocks.

#### Notes on type material.

There is a clear indication in the original description that the latter was based on a single specimen (“Queensland: Kuranda (F.P. Dodd). Type (unique), I. 12698”, which was unambiguously located at SAM and interpreted as holotype. Four specimens from the same locality in ANIC, BMNH and FMNH are labeled variously as “type”, “cotype” or “paratype” and may have been collected with the type, but were evidently not part of the original type series.

### Antimerus smaragdinus

Fauvel, 1878

Figs 11 27–29

Antimerus smaragdinus Fauvel 1878: 550

#### Type locality:

Australia: Victoria: Mountains of Victoria [ca. 36–38°S 146–148°E]

#### Material examined.

**AUSTRALIA: Victoria:** Holotype, pinned, genitalia not dissected, with labels: **“**Mountains of Victoria”, “Antimerus smaragdinus Fauvel, Type” [D. Sharp handwriting], [D. Sharp collection, but this and any other labels not recorded and no FMNH-INS number assigned],1♀ in BMNH. Other material: [no locality], 1♀ in MVMA, 1♂ in ZMHB; Allambee, 6.4 km SSW D/s of W. Tarwin River Falls, 220m, 38°19’S, 146°1’E, 28.ii–19.iii.2000, F.I.T. (N. Porch), 1♂ in Porch; Beech Forest, 11–19.i.1932 (F.E. Wilson), 1♀ in BMNH; Dandenong Ranges, 2♂ in SAM; Mt. Margaret Rd. at Ghost Point, NNE Marysville, 1000m, ANMT 933, 37°27’S, 145°47’E, open Euc. delegatensis forest, 16.ii.1993, FMHD#93-107, berl., leaf & log litter (A. Newton & M. Thayer), 1♀ in FMNH; 16–28.ii.1993, FMHD#93-106, carrion trap (squid) (A. Newton & M. Thayer), 1♂ in FMNH; 16–28.ii.1993, FMHD#93-105, window trap (A. Newton & M. Thayer), 1♀ in FMNH; Mt. Margaret Rd. at Yanks Folly Tr., NNE Marysville, 750m, ANMT 934, 37°28’S, 145°47’E, open Eucalyptus spp. (peppermint & gum) forest, 17.ii.1993, FMHD#93-110, berl., leaf & log litter (A. Newton & M. Thayer), 1♂ in ZMUC; Otway N.P., Binn Rd, 4.3 km N Cape Horn, 390m, ANMT 808, 38°43’S, 143°35’E, wet sclerophyll forest, 25.i.1987, FMHD#87-212, berl., forest litter (A. Newton & M. Thayer), in FMNH; 25.i–8.ii.1987, FMHD#87-210, window trap (A. Newton & M. Thayer), 1♀ in ZMUC; Otway N.P., Elliott R. 5.5 km W Marengo, 80m, ANMT 828, 38°47’S, 143°37’E, wet sclerophyll forest, 8.ii.1987, FMHD#87-262, berl., forest litter (A. Newton & M. Thayer), 4♂, 3♀ in FMNH; Strzelecki S. F., 4.3 km NNE Mirboo North, 205m, 38°37’S, 146°18’E, 16.i–6.ii.2000, F.I.T.-lower (N. Porch), 2♂ in Porch; Tyres R., 5.ii.1966 (M.V.G. Coulson), 1♂ in ANIC; Victorian Alps (E. Fischer), 1♂ in ZMUN; Walsh’s Creek [Victoria?], 24.i.1914, 1♀ in VAIC; Wilson’s Promontory N.P., Lilly Pilly Gully, 15.ii.1990 (R. de Keyzer), 1♀ in AMS; **New South Wales:** Braidwood, Jinden, 3.i.1976 (R.H. Mulder Colln.), in AMS; Brown Mt., 30.xii.1979 (D.P. Carne), 1♂ in ANIC; Brown Mt., 3200 ft [976m], 9.i.1968 (M.S. Upton), in ANIC; Brown Mtn., Canberra [judged in error], 19.ii.1987 (E.A. Sugden), in UCDC; Mt. WogWog (4 km NE), 17 km SE Bombala, 37°4.5’S, 149°28’E, i.1988, pitfalls (C.R. Margules), 1♂ in ANIC; Tuross, 17–22.i.1936 (K.C. McKeown), 1♂ in AMS; Tuross Riv., 11–17.iii.1937 (K.C. McKeown), 1♂ in AMS; **Tasmania:** [no locality] (Sharp Colln.), 1♂ in BMNH; North Head, Pieman R., 29.xii.1953 (I. Rowley), 1♂ in ANIC.

#### Description.

Measurements (n=5): HL: 2.0–2.5; HW: 2.8–3.2; PL: 2.4–3.0; PW: 3.0–3.4; EL: 3.1–3.9; EW: 2.1–3.4. Total length of the body 16–17 mm.

Head, pronotum and elytra metallic green, bluish or purple, abdomen black with slight metallic reflection; often head and pronotum metallic green but elytra with different (bluish or purple) reflection. Disc of head and pronotum without punctuation or pubescence, their surfaces with reticulate microsculpture forming isodiametric mesh. Elytra with dense punctuation and black pubescence, interspaces with reticulate microsculpture. Head, pronotum and elytra somewhat dull, elytra glossy. Appendages brownish-black, mouthparts and apical parts of antennae paler.

Head transverse, considerably wider than long, with rounded but distinct hind angles, tempora slightly shorter than eye (in lateral view). Antennae moderately long, with antennomeres VIII–X about as long as wide, not transverse.

Pronotum slightly wider than long, slightly wider than head, widest along middle third of its length, slightly converging anteriad and posteriad; pronotal anterior angles rounded but distinct, posterior broadly rounded, poorly distinct. Pronotal hypomera not inflexed, visible from lateral view.

Elytron elongate, longer than pronotum.

Wings well developed.

Abdominal tergites IV–VI (second to fourth visible) with shallow transverse impression in basal part, and with vague but distinct midlongitudinal keel-like elevations; tergite VII (fifth visible) with whitish seam at apical margin.

Male (Figs 27–29). Aedeagus with paramere narrowing apically; apical half of the paramere narrower than median lobe, its apex slightly notched (in dorsal, parameral view).

#### Comparison.

Antimerus smaragdinus is most similar to its sister species, Antimerus punctipennis, from which it differs externally in the uniformly colored elytra (no pale humeral spots) and presence of midlongitudinal elevations on the abdominal tergites IV–VI (second to fourth visible). Aedeagi of both Antimerus smaragdinus and Antimerus punctipennis are very similar, the former however having slightly more obtuse apex of the paramere (in dorsal or ventral view).

#### Distribution and bionomics.

Antimerus smaragdinus is confined to the southeastern corner of Australia, including northwestern Tasmania (Fig. 55, A, triangles). Distribution on the mainland covers most of Victoria, the southeast corner of South Australia and southeast corner of New South Wales. It is the only species of the genus which occurs in the south of Australia, being totally allopatric to any other species of Antimerus but abutting the distribution of its sister species Antimerus punctipennis. Specimens of Antimerus smaragdinus with associated bionomic information were collected in wet to dry sclerophyll forests of various types, between 80 and 1000 m elevation by sifting leaf litter, by carrion-baited and unbaited pitfall traps, in flight traps and by hand collecting.

#### Notes on type material.

There is a clear indication in the original description that the latter was based on a single specimen (“Australie méridionale: montagnes de Victoria. – Une seul ♂. Collection Sharp”), which was unambiguously located at the BMNH and interpreted as holotype.

### Antimerus punctipennis

Lea, 1906

Figs 12, 13 30–33

Antimerus punctipennis Lea, 1906: 195.

#### Type locality:

Australia: New South Wales:Gosford [33°26’S, 151°20’E]

#### Material examined.

**AUSTRALIA: New South Wales:** Lectotype (here designated), pinned, genitalia not dissected, with labels:“punctipennis / Lea TYPE Gosford”, “9343 / Antimerus / punctipennis Lea / N. S. Wales / TYPE”,“S. Aust. Museum / specimen” [orange label], “FMNH-INS 0000 019 153”, “LECTOTYPE / Antimerus / punctipennis Lea / A. Solodovnikov des. 2010” [red label], ♂ in SAM. Other material: 1.8km N of ford on Karuah River on Karuah River Rd., Chichester S.F., 32°5’S, 151°43’E, 4.ii–9.iv.1993, 35AM, pitfall trap (M. Gray, G. Cassis), 1♂, 1♀ in AMS; 2.3km N of Karuah River on Karuah River Rd., Chichester S.F., 520m, 32°5’S, 151°43’E, 4.ii–9.iv.1993, 35AR, pitfall trap (M. Gray, G. Cassis), 1♂ in AMS; 240m from jct. of Kunderang East & Kunderang West Rds., 900m, 30°48’S, 152°2’E, 4.ii–9.iv.1993, 39AG, pitfall trap (M. Gray, G. Cassis), 2♀ in AMS; Acacia Plateau, 28°22.8’S, 152°22.2’E (H. Davidson), 1♂ in BMNH; Armidale (40 mi E of), Falls Rd. off Kempsey Rd., 30°36’S, 152°10’E, 6.ii.1965, m.v.l. (C.W. Frazier), 1♀ in ANIC; Batemans Bay, ix.1993 (G.B. Monteith), 1♂ in QM; Beaury S.F. 1/21, N along Wallaby Ck. Rd., 830m, 28°26’S, 152°27’E, 4.ii–9.iv.1993, 01BM, pitfall trap (M. Gray, G. Cassis), 2♂ in AMS; Blackheath (valley near), Blue Mts., 3000 ft [915m], 23.i.1932 (P.J. Darlington), 1♂ in MCZ, 1♀ in FMNH; Blue Mts., 33°30’S, 150°15’E, i.1932 (P.J. Darlington), 2♀ in MCZ; Blue Mts., Wentworth Falls, 33°43’S, 150°22’E, 3.i.1932 (P.J. Darlington), 1♂ in MCZ; 22.i.1963 (D.K. McAlpine), 2♂ in AMS; Booyong, xi.1904 [year “04” but 0 not clear] (Helms Colln.), 1♂ in BPBM; Border Ranges N.P., Brindle Ck., where Brindle Ck. Rd. crosses Brindle Ck., 28°22’S, 153°4’E, 4.ii–9.iv.1993, 40BG, pitfall trap (M. Gray, G. Cassis), 2♀ in AMS; Border Ranges N.P., jct.Tweed Ra.& Brindle Ck. Rds, 1000m, ANMT 787, 28°23’S, 153°6’E, warm-temperate rainfor., 5.i.1987, FMHD#87-170, berl., forest litter (A. Newton & M. Thayer), 1♀ in FMNH; 1–12.i.1987, FMHD#87-168, window trap (A. Newton & M. Thayer), 4♂ in FMNH; Border Ranges N.P., Sheepstation Ck., 530m, ANMT 783, 28°25’S, 153°2’E, subtropical rainforest, 31.xii.1986–12.i.1987, FMHD#86-694, window trap (A. Newton & M. Thayer), 5♂, 1♀ in FMNH; Border Ranges N.P., Tweed Ra.Rd., 0.9 km SE Bar Mt.rd., 1050m, ANMT 785, 28°28’S, 153°8’E, Noth.moorei for. patch, 1–12.i.1987, FMHD#87-160, window trap (A. Newton & M. Thayer), 2♂, 1♀ in FMNH; Border Ranges N.P., Tweed Ra.Rd., 0.9 km SE Bar Mt.rd., 1030m, ANMT 786, 28°28’S, 153°8’E, warm-temperate rainfor., 1–12.i.1987, FMHD#87-165, window trap (A. Newton & M. Thayer), 1♂ in FMNH; Border Ranges N.P., Tweed Range Rd., 4.6km SW of Brindle Ck. Rd., 580m, 28°24’S, 153°2’E, 4.ii–9.iv.1993, 40BM, pitfall trap (M. Gray, G. Cassis), 1♀ in AMS; Branch Ck., 250m downslope from Fife Fire Trail, 2.5km NE of Fife’s Knob Rd., 350m, 30°54’S, 152°23’E, 4.ii–9.iv.1993, 38AG, pitfall trap (M. Gray, G. Cassis), 1♀ in AMS; Broulee, 35°51’S, 150°11’E, i.1969 (W.J.M. Vestjens), 1♀ in ANIC; 9.i.1962 (K.H.L. Key), 1♀ in ANIC; Bulga S.F., Homewoods Rd., 2.8km W of Knodingbul Rd., 690m, 31°37’S, 152°7’E, 4.ii–9.iv.1993, 57CR, pitfall trap (M. Gray, G. Cassis), 1♂ in AMS; Bulga S.F., Pole Bridge Rd., 0.5km E of Knodingbul Rd., 690m, 31°37’S, 152°10’E, 4.ii–9.iv.1993, 57BG, pitfall trap (M. Gray, G. Cassis), 1♂, 2♀ in AMS; Byron Bay, 28°39’S, 153°37’E, xii.1904, 1 in SAM; Carrai S.F. 163A, Fife’s Knob Rd., about 4km from Fife’s Fire Trail, 740m, 30°54’S, 152°22’E, 4.ii–9.iv.1993, 38CG, pitfall trap (M. Gray, G. Cassis), 2♀ in AMS; Carrai SF 163B, Fife’s Knob Rd., about 2km from Fife’s Fire Trail, 800m, 30°54’S, 152°22’E, 4.ii–9.iv.1993, 38CR, pitfall trap (M. Gray, G. Cassis), 2♂ in AMS; Cheltenham, xi–xii.1949 (N.W. Rodd), 1♂ in AMS; Chichester S.F. (nr. Dungog), Hotel Ck.& Bush Mill Rd., 500m, ANMT 777, 32°12’S, 151°43’E, gallery subtrop.rainfor., 16.i.1987, FMHD#87-154, berl., forest litter (A. Newton & M. Thayer), 1♂, 1♀ in FMNH; Currowan S.F. (NW Nelligen), Wallaby For. Pres., 400m, ANMT 771, 35°33’S, 150°2’E, wet sclerophyll forest, 22.xii.1986–18.ii.1987, FMHD#86-658, window trap (A. Newton & M. Thayer), 1♂ in FMNH; Dingo Tops For. Park, Dingo S. F., nw Wingham, 31°39’S, 152°8’E, rainforest, 8.i.1984, rainforest margin (G. Williams), 1♂ in ANIC; Dome Mountain, Richmond Ra. & Yabbra S.F., 600–900m, 28°28’S, 152°43’E, subtropical rainforest sheltered slope, 11.xii.1988, pitfall trap DM3 (Smith, Hines, Pugh & Webber), 1♂ in AMS; Dome Rd., approx. 2km W of Never Never Picnic Area, about 60m up small gully, 710m, 30°21’S, 152°47’E, 4.ii–9.iv.1933, 31AG, pitfall trap (M. Gray, G. Cassis), 2♂ in AMS; Dooragan N.P., Nth Brother Mtn., 450m, 31°39’S, 152°48’E, subtropical rainforest, 29.xii.1998–14.i.1999, malaise (G. Williams), 1♂ in AMS; 25.xi–26.xii.1999, malaise trap (G. & T. Williams), 1♂ in AMS; Dorrigo, 30°19’S, 152°43’E (W. Heron), 1♂, 1 unsexed in SAM, 1♂, 1♀ in ANIC; Dorrigo N.P., 0.8 km NE Park HQ, 740m, ANMT 779, 30°22’S, 152°44’E, subtropical rainforest, 28.xii.1986, FMHD#86-685, berl., forest litter (A. Newton & M. Thayer), 1♀ in FMNH; 28.xii.1986–15.i.1987, FMHD#86-683, window trap (A. Newton & M. Thayer), 2♂ in FMNH; Dorrigo N.P., Wonga Walk, small stream below Tristiana Falls (adjacent to stream), 630m, 30°22’S, 152°43’E, 4.ii–9.iv.1993, 31BG, pitfall trap (M. Gray, G. Cassis), 1♀ in AMS; Dorrigo, 3000 ft [915m], [30°19’S, 152°43’E] (W. Heron), 1 in MCZ; East Kunderang Trail, 1.35km E of West Kunderang Trail, 890m, 30°48’S, 152°2’E, 4.ii–9.iv.1993, 39AM, pitfall trap (M. Gray, G. Cassis), 5♂, 4♀ in AMS; Enfield S.F. 163AS, Daisy Patch Fire Trail, 1.9km S of Enfield Rd., 1130m, 31°20’S, 151°54’E, 4.ii–9.iv.1993, 37BM, pitfall trap (M. Gray, G. Cassis), 1♂ in AMS; Enfield S.F. 163AS, Mummel Forest Rd., 7.6km N of jnct with Enfield Forest Rd., 1340m, 31°17’S, 151°51’E, 4.ii–9.iv.1993, 37AG, pitfall trap (M. Gray, G. Cassis), 4♂, 2♀ in AMS; Ewingar S.F. 2/3, Ewingar Ck., Elkhorn Rd., 640m, 29°5’S, 152°26’E, 4.ii–9.iv.1993, 12AG, pitfall (M. Gray, G. Cassis), 1♂ in AMS; Ewingar SF 11, tributary of Grasstree Ck. Junction with Nogrigar Rd., 720m, 29°8’S, 152°25’E, 4.ii–9.iv.1993, 12BG, pitfall trap (M. Gray, G. Cassis), 8♂ in AMS; Glen Innes (30 km east of), 12.xii.1976 (A. & M. Walford-Huggins), 2♂, 1♀ in BMNH; Helensburgh, 34°10’S, 150°59’E, 4.i.1975, 1♀ in AMS; Intersection of Kunungra & Berrico Rds., Chichester S.F. RM, 1000m, 32°6’S, 151°46’E, 4.ii–9.iv.1993, 35BG, pitfall trap (M. Gray, G. Cassis), 1♂ in AMS; Jenolan Caves, Nat. Park, 33°48’S, 150°1’E, 4.iii.1973 (S. & M. Misko), 1♂ in ANIC; Karuah River crossing, Karuah River Rd., Chichester S.F., 300m, 32°6’S, 151°42’E, 4.ii–9.iv.1993, 35AG, pitfall trap (M. Gray, G. Cassis), 1♀ in AMS; Kempsey (near), McArthurs clearing, 31°4’S, 152°50’E, dry sclerophyll, 4.i.1963 (P. Aitken), 1♀ in SAM; Kioloa S.F., 35°35’S, 150°18’E, rainforest, 4–5.iii.1986, ANIC Berl. 1057, berl., leaf & log litter (J. & N. Lawrence), 1♂ in ANIC; Kioloa S.F., Durras Nth., 35°35’S, 150°18’E, 22.ii.1986 (C. Reid), 1♀ in ANIC; Lansdowne (3 km N), nr. Taree, 31°46’S, 152°32’E, riparian rainforest, 8.xi.1988, in compost on gully rainforest margin (G. Williams), 1♀ in ANIC; 5.xi.1988, in compost on gully rainforest margin (G. Williams), 1♂ in ANIC; Lilyvale, 34°10’S, 151°1’E, 11.iii.1972 [11:3:1972] (D.A. Doolan), 1♀ in AMS; 12.i.1975, 1♂, 1♀ in AMS; London Bridge S.F. 169, approx. 2.7km SW of London Bridge Lookout, 990m, 29°51’S, 152°13’E, 4.ii–9.iv.1993, 27CG, pitfall trap (M. Gray, G. Cassis), 1♂ in AMS; Lorien Wildlife Refuge, 3 km N Lansdowne/Taree, 31°46’S, 152°29’E, rainforest margin, 10–17.i.1988, malaise trap (G. Williams), 1♀ in AMS, 1♀ in ANIC, 1♀ in ZMUC; 1–7.ii.1988, malaise trap (G. Williams), 1♀ in ANIC; 6.i.1987/–/11.i.1987, Samp. 1, malaise trap (G. Williams), 1♀ in AMS; Lumeah Rd., 1.7km from Mt. Allyn Rd., Chichester S.F. 16, 970m, 32°6’S, 151°26’E, 4.ii–9.iv.1993, 36BG, pitfall trap (M. Gray, G. Cassis), 2♂ in AMS; Macksville, [30°43’S, 152°55’E], xii.1990 (Wachtel), 1♀ in NHMW; Macquarie Pass (top), 550m, 34°33’S, 150°38’E, 19.xii.1962 (E.S. Ross, D.Q. Cavagnaro), 1♂ in CAS; Macquarie Pass N.P., Clover Hill Rd., 290m, ANMT 773, 34°34’S, 150°39’E, subtropical rainforest, 23.xii.1986–18.i.1987, FMHD#86-663, window trap (A. Newton & M. Thayer), 1♂, 1♀ in FMNH; Macquarie Pass N.P., NW corner, 490m, ANMT 772, 34°33’S, 150°39’E, subtropical rainforest, 23.xii.1986, FMHD#86-662, berl., forest litter (A. Newton & M. Thayer), 2♀ in FMNH; 23.xii.1986–18.i.1987, FMHD#86-661, carrion trap (squid) (A. Newton & M. Thayer), 1♀ in FMNH; 18.i.1987, flying to shaded rocks, midday (A. Newton & M. Thayer), 1♂ in FMNH, 1♂ in ZMUC; Marengo S.F. 2/3, 0.5km NE along Foamy Ck. Rd. from Chaelundi Rd., 1200m, 30°7’S, 152°24’E, 4.ii.–9.iv.1993, 24BM, pitfall trap (M. Gray, G. Cassis), 3♂, 1♀ in AMS; Marengo S.F. 6/23, 0.4km ENE of jct. of Hardens & Chaelundi Rds., 1290m, 30°8’S, 152°25’E, 4.ii–9.iv.1993, 24BR, pitfall trap (M. Gray, G. Cassis), 1♂ in AMS; Mountain trail, 2.1km S from intersection with Kunungra Rd. on SE side of ford over Kunungra R., 180m, 32°8’S, 151°45’E, 4.ii–9.iv.1993, 35CG, pitfall trap (M. Gray, G. Cassis), 1♂ in AMS; Mt. Allyn Rd., 300m E of Mt. Shellbrook Forest Rd., Chichester SF 46B, 580m, 32°9’S, 151°27’E, 4.ii–9.iv.1993, 36AG, pitfall trap (M. Gray, G. Cassis), 5♂, 1♀ in AMS; Mt. Boss S.F. 163A, N Plateau Rd., 3.5km from Plateau Beech Picnic Area, 1120m, 31°11’S, 152°20’E, 4.ii–9.iv.1993, 32BR, pitfall trap (M. Gray, G. Cassis), 1♀ in AMS; Mt. Boss S.F. 17, N Plateau Rd., 1.5km by track from Plateau Beech Picnic Area, 1120m, 31°10’S, 152°19’E, 4.ii–9.iv.1993, 32BM, pitfall trap (M. Gray, G. Cassis), 3♂ in AMS; Mt. Boss S.F. 17, small gully near Plateau Beech Picnic area, end of Plateau Rd., 1040m, 31°10’S, 152°19’E, 4.ii–9.iv.1993, 38BG, pitfall trap (M. Gray, G. Cassis), 2♂, 1♀ in AMS; Mt. Boss S.F. 2, Rimau Rd., about 13.8km E of Cockera Wombeeba Rd., 760m, 31°11’S, 152°22’E, 4.ii–9.iv.1993, 32CR, pitfall trap (M. Gray, G. Cassis), 1♂ in AMS; Mt. Boss S.F. 47, Rimau Rd., about 11.2km E of Cockera Wombeeba Rd., 690m, 31°11’S, 152°21’E, 4.ii–9.iv.1993, 32CM, pitfall trap (M. Gray, G. Cassis), 1♀ in AMS; Mt. Corricudgy, 1200m, 32°50’S, 150°21’E [in error on label as 30°50’S, 149°43’E], forest, 12.xii.1982, E-Y AU 39, beaten in forest (S. Endrödy-Younga), 1 in TMSA; 11.xii.1982, E-Y AU 38, logs and bark (S. Endrödy-Younga), 1 in TMSA; Mt. Hyland N.R. 3/11, 0.9km S along Chaelundi Rd. from Big Bull Ck. Rd., 1080m, 30°9’S, 152°27’E, 4.ii–9.iv.1993, 24AR, pitfall trap (M. Gray, G. Cassis), 8♂, 5♀ in AMS; Mt. Hyland N.R. 3/11, 1.9km N along Chaelundi Rd. from Big Bull Ck. Rd., 1160m, 30°8’S, 152°26’E, 4.ii–9.iv.1993, 24AM, pitfall trap (M. Gray, G. Cassis), 1♂, 2♀ in AMS; Mt. Hyland N.R. 3/11, Obeloe Ck., 2.0km SW along Obeloe Rd. from Chaelundi Rd., 910m, 30°9’S, 152°27’E, 4.ii–9.iv.1993, 24AG, pitfall trap (M. Gray, G. Cassis), 25♂, 10♀ in AMS, 1♂ in FMNH; Mt. Hyland N.R., NW of Dorrigo, 30°9’S, 152°26’E, cool temperate rainforest, 25.i.1996, under log (G. & B. Williams), 1♀ in AMS; Mt. Hyland N.R., Obeloe Rd., NW of Dorrigo, 30°9’S, 152°26’E, margin of subtropical rainforest, 25.i.1996 (G. & B. Williams), 1♂ in AMS; NE facing slope above Kunderang Station Ck., 410m, 30°48’S, 152°6’E, 4.ii–9.iv.1993, 39BR, pitfall trap (M. Gray, G. Cassis), 2♂ in AMS; New England N.P., 30°29’S, 152°30’E, woodland, 25.i.1979, on rat carcass (S.A. Harrington), 1♀ in ANIC; New England N.P. 17, Cliffs Trail (top end), about 3km S of Point Lookout, 1350m, 30°31’S, 152°23’E, 4.ii–9.iv.1993, 33AM, pitfall trap (M. Gray, G. Cassis), 2♂; New England N.P., Cliffs Trail (top end), 1350m, 30°30’S, 152°23’E, 4.ii–9.iv.1993, 33AR, pitfall trap (M. Gray, G. Cassis), 16♂, 2♀ in AMS; New England N.P., Cliffs Trail about 2km S of gate from Pt. Lookout Rd., 1300m, 30°30’S, 152°23’E, 4.ii–9.iv.1993, 33AG, pitfall trap (M. Gray, G. Cassis), 7♂, 1♀ in AMS; New England N.P., Toms Cabin, 30°30’S, 152°24’E, 12–24.ii.1984, Malaise trap (I.D. Naumann), 1♀ in ANIC; Nightcap N.P., Mt. Nardi, Newton Dr., 700m, ANMT 791, 28°33’S, 153°17’E, warm-temperate rainfor., 4.i.1987, FMHD#87-179, berl., forest litter (A. Newton & M. Thayer), 2♂, 1♀ in FMNH; O’Sullivan’s Gap F. P., VWHL-216, 32°21’S, 152°15’E, wet sclerophyll forest, flood refuse (V.W.H. Lorimer), 1♂, 1♀ in Lorimer; Ramornie S.F. 74A, Track off Mt. Tindal Rd., 200m, 29°42’S, 152°38’E, 4.ii–9.iv.1993, 20BM, pitfall trap (M. Gray, G. Cassis), 3♂ in AMS; Richmond Range S.F., Cambridge Plateau, 300–600m, 28°47’S, 152°45’E, subtropical rainforest exposed slope, 17.xii.1988, Pitfall trap CP6 (Smith, Hines, Pugh & Webber), 1♂ in AMS; Richmond R., 28°45’S, 153°0’E, 1♂, 1♀ in BMNH; Royal N.P., Palm Gully, off Lady Wakehurst Dr., 80m, ANMT 774, 34°9’S, 151°2’E, subtropical rainforest, 25.xii.1986–17.i.1987, FMHD#86-667, window trap (A. Newton & M. Thayer), 1♂ in FMNH; Solferino Ck., track off Lionsville Rd., Ewingar S.F., 520m, 29°10’S, 152°26’E, 4.ii–9.iv.1993, 12CG, pitfall trap (M. Gray, G. Cassis), 1♂ in AMS; Styx River S.F. 163 & 17, off Cunnawarra Trail, Cunnawarra Ck. (800m N), 950m, 30°33’S, 152°19’E, 4.ii–9.iv.1993, 33CG, pitfall trap (M. Gray, G. Cassis), 2♂ in AMS; Styx River S.F. 163, bottom end of Cliffs Trail, Oxley Rd. (about 1.3km NE), 1080m, 30°33’S, 152°20’E, 4.ii–9.iv.1993, 33BR, pitfall trap (M. Gray, G. Cassis), 1♂, 1♀ in AMS; Styx River S.F. 163, off Cunnawarra Trail, 1130m, 30°32’S, 152°20’E, 4.ii–9.iv.1993, 33CR, pitfall trap (M. Gray, G. Cassis), 2♂, 1♀ in AMS; Styx River S.F. 17, bottom end of Cliffs Trail, Oxley Rd. (about 2.8km NE), 1130m, 30°33’S, 152°21’E, 4.ii–9.iv.1993, 33BM, pitfall trap (M. Gray, G. Cassis), 1♀ in AMS; Swerly branch of Kunderang Station Ck., 310m, 30°48’S, 152°6’E, 4.ii–9.iv.1993, 39BG, pitfall trap (M. Gray, G. Cassis), 3♂, 2♀ in AMS; ‘Tuglo’, 48 km N of Singleton (32 33 151 10), 32°10’S, 151°10’E, i.1977, pit trap (M. Gray), 1♂ in AMS; Tweed Range, 800–900m, 28°32’S, 153°16’E, subtropical rainforest, 21.ii.1989, pitfall trap T2.52 (Smith, Hines, Pugh & Webber), 1♂ in AMS; Unumgar S.F. (nr. Grevillia), Coxs Rd., 580m, ANMT 789, 28°27’S, 152°45’E, subtropical rainforest, 2–11.i.1987, FMHD#87-175, window trap (A. Newton & M. Thayer), 1♂ in FMNH; Urbenville-Legume Rd, gully in middle of Toolom Scrub flora reserve, 715m, 28°28’S, 152°23’E, 4.ii–9.iv.1993, 01AG, pitfall trap (M. Gray, G. Cassis), 1♀ in AMS; Washpool N.P., Cedar Ck., Cedar Trail, 920m, 29°28’S, 152°20’E, 4.ii–9.iv.1993, 17CG, pitfall trap (M. Gray, G. Cassis), 2♂, 3♀ in AMS; Washpool N.P., track off Cedar Trail, 890m, 29°28’S, 152°20’E, 4.ii–9.iv.1993, 17CR, pitfall trap (M. Gray, G. Cassis), 1♂ in AMS; Washpool N.P., track off Cedar Trail, 950m, 29°28’S, 152°20’E, 4.ii–9.iv.1993, 17CM, pitfall trap (M. Gray, G. Cassis), 21♂, 3♀ in AMS; Washpool S.F., 29°19’S, 152°24’E, 22.ii–9.iii.1992, FN 5081, pit trap; trap 2 (M. Gray & P. Croft), 1♀ in AMS; Washpool S.F., Coombadjah, 29°30’S, 152°19’E, 9.ii.1982, pit trap (C. Horseman), 1♂ in AMS; Wild Cattle Creek S. F., 30°14’S, 152°45’E, wet sclerophyll forest, 29.iii–30.v.1993, VWHL-381, pitfall trap (bait: human dung) (V.W.H. Lorimer), 1♀ in Lorimer; Wingham, 31°51’S, 152°22’E, 1933, 1♀ in AMS; Wonga Walk, about 600m N of Tristiana Falls, Dorrigo N.P., 730m, 30°22’S, 152°43’E, 4.ii–9.iv.1993, 31BM, pitfall trap (M. Gray, G. Cassis), 3♂, 2♀ in AMS; Wonga Walk, nr. Hardwood Lookout, about 200m SW of Hardwood Lookout, Dorrigo N.P., 630m, 30°22’S, 152°44’E, 4.ii–9.iv.1993, 31BR, pitfall trap (M. Gray, G. Cassis), 3♂ in AMS; Woronora Dam Catchment, Fire Rd. No. 9, 34°11’S, 150°54’E, 8–12.xii.1999, HS STH SYD. -12, pitfalls (M. Gray, G. Milledge & H. Smith), 1♂ in AMS; Yabbra S.F., Yabbra Scrub, 300–600m, 28°38’S, 152°30’E, dry subtropical rainforest, sheltered gully, 14.xii.1988, pitfall trap Y6 (Smith, Hines, Pugh & Webber), 1♂ in AMS; **Queensland:** Joalah Nat. Park, rainforest, 380 m, 14.iii.1973, 1♀ in ZMUC; 27°55’S, 153°12’E Cunningham’s Gap, 790m, 28°3’S, 152°24’E, rainforest, 6.i–1.iii.1992, intercept (D.J. Cook), 1♀ in QM; Fletcher, S. Q., 20.xi.1965 (E. Fulton), in QM; Lamington N.P., 6–10.ii.1961 (F.A. Perkins), 1♀ in UQIC; 28.i–3.ii.1963 (G. Monteith), 1♀ in UQIC; Lower Coomera, 350m, 28°11’S, 153°11’E, 9.i–6.iv.1995, intercept trap (G. Monteith), 1♂, 1♀ in QM; 3.xii.1994–9.i.1995, pitfall trap (G. Monteith & H. Janetzki), 1♀ in QM; Mistake Mtns. (Middle), via Goomburra, 950m, 1976–1977, pitfall 75 (G.B. & S.R. Monteith), 1♂, 2♀ in QM; Montville (C. Deane), 1♀ in UQIC; Mt. Barney, 29.xii.1961 (J. Bryan), 1♂ in UQIC; Mt. Glorious, 27°19.9’S, 152°45.48’E, rainforest, 5.i.1974, pitfall No. 1, 1♂ in UQIC; Mt. Glorious, 27°19.9’S, 152°45.48’E, 10–31.i.1982, Malaise trap (T. Hiller), 1♂ in QDPC; Mt. Huntley, 1260m, 28°8’S, 152°26’E, 20.xii.1992–??.iii.1993, intercept & pitfall (G. Monteith), 1♂ in QM; Mt. Superbus Summit, 1300m, rainforest, 8.ii–12.iii.1990, pitfall traps (Monteith, Thompson & Janetzki), 1♂ in QM; Mt. Tambourine, 27°55’S, 153°10’E, 2.i.1972 (P. Allsopp), 1♂ in QDPC; National Park, xii.1921 (H. Hacker), in QM; Springbrook Repeater, 1000m, 28°15’S, 153°16’E, Rf, 21.xii.1996 (G.B. Monteith), 1♂ in QM; Sunday Ck., Conondale Ra., 900m, 26°43’S, 152°34’E, rainforest, 29.xi.1991–7.i.1992, intercept (D.J. Cook), 1♂ in QM; Tambourine Mt., 27°55’S, 153°10’E (C. Deane), 1♀ in UQIC; Upper Cedar Ck., via Samford, 3.i.1963 (T. Brooks), 1♂ in BPBM.

#### Description.

HW: 3.1–3.9; PL: 2.9–3.5; PW: 3.1–3.7; EL: 3.4–4.1; EW: 3.6–4.2. Total length of the body 16–18 mm.

Head, pronotum and elytra metallic green, brassy or metallic purplish, abdomen dark brown to black, without strong metallic reflection, often with pale brown apical margin of tergite VII (fifth visible) and (or) pale brown apical part of tergite IV (second visible); humeri of elytra laterally (often) and stripe along elytral suture (seldom) pale brown; appendages brown. Disc of head and pronotum without punctuation or pubescence, their surfaces with reticulate microsculpture forming isodiametric mesh. Elytra with dense punctuation and black pubescence, interspaces with weak irregular (not isodiametric) microsculpture. Head, pronotum and elytra somewhat dull, elytra glossy.

Head considerably wider than long, with rounded but very distinct hind angles, tempora slightly shorter than eye (in lateral view). Antennae moderately long, with antennomeres VIII–X about as long as wide, not transverse.

Pronotum slightly wider than long, about as wide as head; pronotal anterior angles rounded but distinct, posterior broadly rounded, poorly distinct; pronotum widest along middle third of its length, slightly converging anteriad and posteriad. Pronotal hypomera not inflexed, visible from lateral view.

Elytron elongate, longer than pronotum.

Wings well developed.

Abdominal tergites IV–VI (second to fourth visible) with very shallow transverse impression in basal part; tergite VII (fifth visible) with whitish seam at apical margin. Abdominal terga without vague longitudinal elevation along midline.

Male (Figs 30–33). Aedeagus with paramere narrowing apically, apical half of the paramere narrower than median lobe (in dorsal or ventral view); paramere slightly notched at the apex.

#### Comparison.

Antimerus punctipennis is most similar to its sister species, Antimerus smaragdinus, from which it differs externally in the presence of brownish humeral spots on the elytra and absence of vague midlongitudinal elevations on abdominal tergites IV–VI (second to fourth visible). Aedeagi of both species are very similar, the former however having a slightly more pointed apex of the paramere (in dorsal or ventral view, cf. Figs 32 and 29). For differences of Antimerus punctipennis from Antimerus metallicus, another very similar species, see the latter below.

#### Distribution and bionomics.

Antimerus punctipennis is the most common species of the genus, distributed in eastern Australia from the south of Queensland to the south of New South Wales. In the southeastern corner of New South Wales its distribution abuts that of Antimerus smaragdinus (Fig. 55, A, squares – Antimerus punctipennis, triangles – Antimerus smaragdinus), but these sister species are apparently completely allopatric. All adult specimens of Antimerus punctipennis were collected in various types of temperate to tropical forests, especially in rainforests but also in wet to dry sclerophyll forests, at elevations from 80 to 1350 m, mostly on the ground, by pitfall traps or by sifting leaf litter. Some specimens came to flight intercept traps (e.g., Fig. 56), Malaise traps, or were hand collected from logs and by beating tree branches; two were collected while flying to shaded rocks at the edge of a clearing at midday. All presumed larvae of this species (see below) were collected from forest leaf litter. The available sample suggests that Antimerus punctipennis is mostly confined to living on the ground with the ability to climb tree trunks and possibly branches, or other vegetation.

#### Notes on lectotype designation.

In the original description of Antimerus punctipennis there is no clear indication about the number of specimens examined; the type locality and collector (“Gosford, N.S.W., A.M. Lea”) is the only information available from this description. To unambiguously fix the identity of this species, a male syntype from Gosford in SAM is here designated as lectotype. This is the only specimen we have seen from this locality in SAM or elsewhere, so we have not been able to recognize any paralectotypes, although Lea (1906: 195) stated “I have seen this species in several collections under the name of Antimerus smaragdinus, and had it so named myself ….”

### Antimerus metallicus sp. n.

urn:lsid:zoobank.org:act:CF3AC93C-CC69-4136-A619-04795498A123

Figs 14 30–32, 34 

#### Type locality:

Australia: Queensland: Longlands Gap,1150m, 17°28’S, 145°29’E

#### Material examined.

**AUSTRALIA: Queensland:** Holotype, pinned, genitalia not dissected but aedeagus partially protruding from abdomen, with labels:“17.28S 145.29E QLD / Longlands Gap BS1 / 1150m 6 Mar–4 Apr **/** 1995 P. Zborowski / FI Traps”, “FMNH-INS 0000 019 119”, “HOLOTYPE / Antimerus / metallicus sp. n. / A. Solodovnikov des. 2006” [red label], ♂ in ANIC. Paratypes:Atherton, 17°16’S, 145°28’E, 26.i.1962 (G.W.S.), 1♂ in QPIM, 1♀ in QDPC; Atherton Tab., Mts. above (SW) Millaa Millaa, c. 3500 ft [1067m], xii.1957 (Darlingtons), 1♂ in FMNH, 1♀ in MCZ; Baldy Mtn. Rd., 7 km SW Atherton, 1150m, 9.xii.1988 (Monteith & Thompson), 1♀ in QM; Baldy Mtn. via Atherton, MDPI F.I.T. site 37, 5.xi–10.xii.1992, F.I.T. (S. De Faveri & R. Storey), 1♂ in QPIM; 5.xii.1975, leaf litter (R. Storey), 1♂ in QPIM; Bally Knob summit, 1100m, [M-C] 2147, 17°39’S, 145°30’E, open for., 6.xii.1998–6.ii.1999, pitfall (Monteith & Cook), 1♂ in QM; Bellenden Ker, 10.i.1977 (A. & M. Walford-Huggins), 1♂ in BMNH; Bones Knob (3 km W), 1100m, 17°13’S, 145°25’E, 10.xii.1995 (Monteith, Cook & Thompson), 1♀ in QM; Bones Knob (3 km W), 1140m, 17°14’S, 145°25’E, RF, 10.xii.1995–9.ii.1996, intercept trap (Monteith, Cook & Thompson), 1♂ in QM; Davies Ck., 22 km WSW of Mareeba, 6.xi–2.xii.1984, Malaise T. (Storey & Halfpapp), 1♂ in QPIM; Herberton (4 mi NE), Herberton Ra., 4000 ft [1220m], rainforest, 8.xi.1966, under logs (E. Britton), 1♂ in ANIC; Hinchinbrook I., Upper Gayundah Ck., 850m, R.F., 9–11.xi.1984 (G. Monteith & D.J. Cook), 2♂ in QM; Hugh Nelson Range, 21 km S Atherton, [RIS] Site 17, 17°27.55’S, 145°28.35’E, 1.xii.1983–9.i.1984, FIT (Storey & Brown), 1♂, 1♀ in FMNH, 1 sex unknown in QPIM, 1♂ in ZMUC; 9.i–10.ii.1984, FIT (Storey & Brown), 2♂ in FMNH, 1♂ in QPIM; Hugh Nelson Range, 1150 m, flight intercept trap, 1.xi – 3.i.1995, leg. P. Zborowski, 1♂ in ZMUC; Kirrama Rge., via Cardwell, 2–3000 ft, ii.1958 (Darlingtons), 1♂, 1♀ in MCZ; Kuranda (6 km SE), MDPI FIT site 20, 16°51.98’S, 145°36.98’E, 15.i–20.ii.1985, FIT (Storey & Halfpapp), 6♂, 2♀ in FMNH, 1♂, 1 sex unknown in QPIM; Maalan SF on Hwy., 850m, 17°35’S, 145°35’E, 25.xi.1994–10.i.1995, flt. intercept trap (Monteith & Hasenpusch), 1♂ in QM; Massey Ck., 17°37’S, 145°34’E 1000m, BS3, 1.xii.1994–3.i.1995, FI Trap JCU (East) (P. Zborowski), 1♂ in ANIC, 1♂ in ZMUC; FI Trap JCU (West), 1♂ in ANIC; FI Trap ANIC, 1♂ in ANIC; 3.i–5.ii.1995, FI Traps, 1♂, 1♀ in ANIC; 3.ii–6.iii.1995, pitfall traps, 1♀ in ANIC; 6.iii–5.iv.1995, FI Traps, 1♂ in ANIC; 30.xi.1995–3.i.1996, pitfall traps (L. Umback), 1♂, 2♀ in ANIC; 2–30.xi.1995, pitfall traps, 1♀ in ANIC; 3–31.i.1996, 1♀ in ANIC; Mt. Fisher, 1150m, 17°33’S, 145°32’E, 3.i–4.ii.1995, BS2, F I Trap JCU (East) (P. Zborowski), 1♀ in ANIC; Mt. Haig, 1150m, 17°06’S, 145°36’E, 1.xii.1994–3.i.1995, GS1, F I Trap JCU (East) (P. Zborowski), 1♂ in ANIC; Mt. Spec, 880m, 18°55’S, 146°10’E, 4.xi–1.xii.1995, S2, F I Trap JCU (M. Cermak), 1♂ in ANIC; 10.i–6.ii.1995, F I Traps (M. Cermak), 1♀ in ANIC; Mt. Spurgeon (2 km SE), via Mt. Carbine, 1100m, RF, 20.xii.1988–4.i.1989, flt. intercept (Monteith, Thompson & ANZSES), 1♂ in QM; Paluma (c. 4 km W), 12.xii.1972 (J.G. Brooks), 1♂ in ANIC; Ravenshoe (9 km N), 1060m, [M-C] 2137, 17°32’S, 145°29’E, wet sclerophyll, 6.xii.1998–5.ii.1999 (Monteith & Cook), 1♂ in QM; Townsville, 1♂ in BMNH; Tully Falls S.F. via Ravenshoe, 20.xi.1977, ex leaf litter (R. Storey, N. Gough), 2♀ in QPIM; Tully Falls S.F., 9.5 km SSW Ravenshoe, 1000m, MDPI site 29A, 17°41.23’S, 145°30.9’E, 9.ii–3.iii.1988, FIT (Storey & Dickenson), 1♀ in FMNH; 5.xi–7.xii.1987, intercept trap (Storey & Dickenson), 1♂ in QPIM; Windsor Tableland, 42 km from highway, MDPI FIT site 14a, 16°15.22’S, 145°2.27’E, 10.xi–26.xii.1983, FIT (Storey & Watford-Higgins), 1♀ in FMNH.

#### Description.

Measurements (n=5): HL: 2.0–2.4; HW: 3.0–3.5; PL: 2.5–3.1; PW: 2.8–3.3; EL: 3.2–3.9; EW: 3.6–4.2. Total length of the body 16–18 mm.

Head and pronotum metallic green with brassy reflection, or the reverse; coloration of elytra very variable, from mostly dark metallic green on disc with only brownish epipleura, through brownish with more or less large dark metallic spot on disc, to entirely brownish elytra, without any dark coloration; abdomen dark brown to black, with only weak metallic reflection, with brown-reddish apical margins of tergites, tergite VIII (sixth visible) being brown-reddish in its apical half; legs dark brown, antennae paler. Disc of head and pronotum without punctuation or pubescence, their surfaces with microsculpture of transverse waves. Elytra with dense punctuation and brown pubescence, interspaces with weak irregular microsculpture. Head and pronotum glossy, with strong metallic reflection, elytra and abdomen less glossy.

Head considerably wider than long, with broadly rounded hind angles, tempora distinctly shorter than eye (in lateral view). Antennae moderately long, with antennomeres VIII–X about as long as wide, not transverse.

Pronotum slightly wider than long, only slightly narrower than head; pronotal anterior angles rounded but distinct, posterior angles also distinct although more rounded; pronotum widest before (anterior to) its middle, converging anteriad and posteriad. Pronotal hypomera not inflexed, visible from lateral view.

Elytron elongate, longer than pronotum.

Wings well developed.

Abdominal tergites IV–VI (second to fifth visible) with moderately deep transverse impression in basal part and slightly elevated obtuse median keels across these impressions; tergite VII (fifth visible) with whitish seam at apical margin.

Male (Figs 30–32, 34). Aedeagus with paramere narrowing apically, apical half of the paramere narrower than median lobe (in dorsal or ventral view); paramere slightly notched at the apex.

#### Comparison.

Antimerus metallicus is very similar to Antimerus punctipennis and Antimerus smaragdinus, but differs well from both of them in having transverse wavy (rather than reticulate) microsculpture of the disc of the head and pronotum, these bodyparts therefore appearing more glossy. The shape of the aedeagus in Antimerus metallicus is practically identical to that of Antimerus punctipennis (hence, Figs 30–32 can serve for both) except for a subtle difference in the shape of the sclerotized piece of the internal sac (cf. Figs 33 and 34).

#### Distribution and bionomics.

Antimerus metallicus is confined to northern Queensland (Fig. 55, A, circles). Its distribution is well separated from those of the closely related Antimerus punctipennis and Antimerus smaragdinus. Adults of Antimerus metallicus were collected in rainforest and wet sclerophyll forest, mostly in highlands between 700 and 1300 m elevation, and mostly by pitfall traps or by flight intercept traps. Some specimens, including two presumed larvae, were sifted from leaf litter, some were hand collected from the ground, and one specimen came to a Malaise trap.

#### Etymology.

The name of the new species is from the Latin adjective *metallicus*, referring to the metallic reflection of its body coloration.

### Antimerus jamesrodmani sp. n.

urn:lsid:zoobank.org:act:406985AC-8A7D-4FA1-B4FF-9BD00233CB5B

Figs 16 35–37 

#### Type locality:

Australia: Queensland:Mt. Glorious State Forest (NW of Brisbane), 750m, 27°23’S, 152°45’E

#### Material examined.

**AUSTRALIA: Queensland:** Holotype, pinned, aedeagus dissected and attached to the specimen in a plastic genitalia vial with glycerin; with labels “AUSTRALIA: Qld.,/ Mt. Glorious St. For./ (nw Brisbane), 750m,/ subtropical rainforest/ Y. Basset canopy study”, “ex canopy (25m) of/ Agyrodendron actinophyllum/ Edlin (Sterculiaceae)”, “12-19.II.87/ IT3 (OFT)”, “FMNH-INS 0000 019 164”, “HOLOTYPE Antimerus jamesrodmani sp. nov. A. Solodovnikov des. 2008”, ♂ in QM. Paratypes: same locality, 11–18.xii.1986, ex canopy (25m) Agyro. actinoph., IT1 (0FT) (Y. Basset), FMNH-INS 0000 019 172, 1♂ in ZMUC; same locality, 12–19.ii.1987, ex canopy (25m) Agyro. actinoph., IT3 (0FT) (Y. Basset), FMNH-INS 0000 019 165, 1♂ in FMNH; same locality, 2–8.ii.1987, ex canopy (25m) Agyro. actinoph., IT4 (0FT) (Y. Basset), FMNH-INS 0000 019 166, 1♂ in ZMUC; same locality, 25.xii.1986–2.i.1987, ex canopy (25m) Agyro. actinoph., IT5 (0FT) (Y. Basset), FMNH-INS 0000 019 163, 1♀ in FMNH.

#### Description.

Measurements (n=4): HL: 1.7–2.2; HW: 2.5–2.9; PL: 2.4–2.7; PW: 2.7–3.0; EL: 3.0–3.5; EW: 3.0–3.5. Total size of the body 14 –16 mm.

Head and pronotum metallic blue with purple reflection, very glossy; elytra red with metallic reflection; abdomen dark brown to black, with weak metallic reflection, antennae and tarsi paler, brown. Disc of head and pronotum without punctuation or pubescence, their surfaces with microsculpture of transverse waves and micropunctuation. Elytra with sparse punctuation bearing brown to black pubescence, interspaces without distinct microsculpture. Abdomen moderately densely punctuated, with brown to black pubescence.

Head wider than long; tempora tapered towards relatively narrow neck, forming broadly rounded, poorly distinct hind angles, about as long as eye (in lateral view); neck delimited from head dorsally by very fine groove. Antennae with antennomeres VIII–X distinctly wider than long, transverse.

Pronotum slightly wider than long, as wide as head; pronotal anterior and posterior angles rounded but distinct; pronotum widest in its middle, converging more strongly anteriad than posteriad. Pronotal hypomera inflexed, not visible from lateral view except for its translucent postcoxal process.

Elytron elongate, longer than pronotum.

Wings well developed.

Abdominal tergites IV–VI (second to fourth visible) with moderately deep transverse impression in basal part; tergite VII (fifth visible) with whitish seam at apical margin.

**Figures 35–39.**
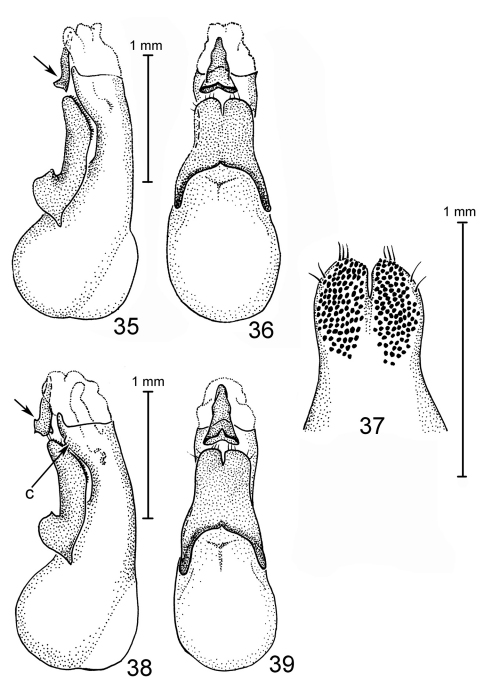
Species of Antimerus, aedeagi: **35–37** *jamesrodmani* **38, 39** *bellus* **35, 38** aedeagus laterally **36, 39** aedeagus dorsally (parameral side) **37** apical portion of paramere, underside; *c*, carina; arrows point to the sclerite of the internal sac.

Male (Figs 35–37). Aedeagus with broad paramere, which is as wide as median lobe (in dorsal or ventral view); parameral apex distinctly bilobed, lobes separated by deep narrow incision.

#### Comparison.

Antimerus jamesrodmani is most similar to Antimerus bellus and Antimerus gracilis. From both of them Antimerus jamesrodmani differs in having red elytra and the neck delimited from the head dorsally by very fine groove (rather than not delimited). From Antimerus bellus additionally it differs in having slightly more distinct hind angles of the head. Aedeagi of Antimerus jamesrodmani and Antimerus bellus are very similar and differ slightly in the shape of the sclerotized piece of the internal sac. Also, unlike that in Antimerus bellus, the median lobe in Antimerus jamesrodmani lacks lateral carinae on its parameral side near apex (in lateral view; cf. Figs 35 and 38, carina indicated by “c”; sclerotized piece indicated by arrow).

#### Distribution and bionomics.

Antimerus jamesrodmani is known only from the type locality in southeastern Queensland (Fig. 55, B, triangle). All known specimens were collected in subtropical rainforest from the canopy of the tree Argyrodendron actinophyllum Edlin (Sterculiaceae) 25 m above ground (see Basset 1991), suggesting an arboreal life style for this species.

#### Etymology.

It is our pleasure to dedicate this noticeable rove beetle species to Dr. James Rodman, who promoted and then, for a number of years, managed the “Partnership of Enhancing Expertise in Taxonomy” (PEET) program at the National Science Foundation of the United States. The PEET funding greatly facilitates systematic exploration of poorly known groups of organisms on a world basis and provides unique opportunities for young systematists to develop their own careers. In particular, our taxonomic exploration of the poorly known Australian Staphylinidae greatly benefited from the PEET program.

### Antimerus bellus sp. n.

urn:lsid:zoobank.org:act:16D3A62E-1BE8-41B5-9C3B-64B925010BAC

Figs 17 38, 39 

#### Type locality:

Australia: New South Wales: Royal National Park, near Sydney [ca. 34°07’S, 151°02’E]

#### Material examined.

**AUSTRALIA: New South Wales:** Holotype, pinned, aedeagus dissected and attached to the specimen in a plastic genitalia vial with glycerin; with labels: **“**Royal National Park/ near Sydney, NSW/ 12 Feb 1985/ D.K. McAlpine/ B.J. Day)”, “Antimerus/ det/ VWH Lorimer/ Oct 96”, “AMSA”, “Antimerus/ n. sp.5/ det. A. Newton 2005”, “HOLOTYPE/ Antimerus/ bellus sp.n./ A. Solodovnikov des. 2006”, “FMNH-INS/ 0000 019 143”, ♂ in AMS. Paratype: Glen Innes, Prison Farm, vii.1969–xii.1970 (“coll.?”), FMNH-INS 0000 019 094, 1♀ in ANIC.

#### Description.

Measurements (n=2): HL: 1.7–2.2; HW: 2.5–2.9; PL: 2.4–2.7; PW: 2.7–3.0; EL: 3.0–3.5; EW: 3.0–3.5. Total size of the body 14–16 mm.

Head, pronotum and elytra metallic blue with purplish reflection, very glossy; abdomen and appendages dark brown. Disc of head and pronotum without punctuation or pubescence, their surfaces with microsculpture of transverse waves and faint micropunctuation. Elytra with sparse punctuation and brown to black pubescence, interspaces without distinct microsculpture. Abdomen moderately densely punctuated, without distinct metallic reflection, with brown to black pubescence.

Head wider than long, with tempora strongly tapered towards relatively narrow neck, about as long as eye (in lateral view); neck not delimited from head dorsally. Antennae with antennomeres VIII–X distinctly wider than long, transverse.

Pronotum slightly wider than long, as wide as head; pronotal anterior and posterior angles rounded but distinct; pronotum widest behind its middle in the area of its posterior angles, its sides, from posterior angles to middle very gradually and anterior from middle, more strongly, converging anteriad. Pronotal hypomera inflexed, not visible from lateral view except for its translucent postcoxal process,.

Elytron elongate, longer than pronotum.

Wings well developed.

Abdominal tergites III–V (first to third visible) with moderately deep transverse impression in basal part; tergite VII (fifth visible) with whitish seam at apical margin.

Male (Figs 38, 39). Aedeagus with paramere as wide as median lobe (in dorsal or ventral view), distinctly bilobed; lobes separated by deep narrow incision.

#### Comparison.

Antimerus bellus is most similar to Antimerus jamesrodmani and Antimerus gracilis. From Antimerus jamesrodmani it differs in the shape of the head which has more broadly rounded, indistinct hind angles, and in the coloration of the elytra, which are not red and have a stronger metallic reflection similar to that of head and pronotum. Aedeagi of Antimerus bellus and Antimerus jamesrodmani are very similar; for differences see “comparison” under the latter species . From Antimerus gracilis, Antimerus bellus differs in coloration (cf. Figs 17 and 18) of the body.

#### Distribution and bionomics.

Antimerus bellus is known only from two specimens collected in different localities in eastern New South Wales (Fig. 55, B, circles). No habitat data are available.

#### Etymology.

The name of the new species is from the Latin adjective *bellus*, or beautiful.

### Antimerus gracilis sp. n.

urn:lsid:zoobank.org:act:642AE774-3ED6-4B55-89A5-A69C2C283E42

Fig. 18 

#### Type locality:

Australia: Queensland:Bellenden Ker, 1994 Crash site, 1325m, 17°16’S, 145°51’E

#### Material examined.

**AUSTRALIA: Queensland:** Holotype, pinned, genitalia not dissected, with labels: “NEQ17°16’S X 145°51’E / Bellenden Ker, 1994/ Crash site. 1 Dec. 1998/ G. Monteith. Pyrethrum/ trees. 1325m 1992”, “QUEENSLAND/ MUSEUM LOAN/ DATE: Sept. 2004/ No. LE 04.51”, “FMNH-INS/ 0000 019 173”, “Antimerus/ n. sp.4/ det. A. Newton 2004”, “HOLOTYPE/ Antimerus/ gracilis sp.n./ A. Solodovnikov des. 2006” , ♀ in QM.

#### Description.

Measurements (holotype): HL: 1.8; HW: 2.6; PL: 2.4; PW: 2.5; EL: 3.1; EW: 3.3. Total length of the body: 15 mm.

Head and pronotum metallic green, with slight brassy reflection, very glossy; elytra, on disc dark brown with bluish metallic reflection, but at shoulders, in epipleural areas, along suture and apical margins brown, without metallic reflection; abdomen dark brown except for orange apex (apical part of segment VII and entire segment VIII (fifth and sixth visible) orange); legs dark brown; antennae paler. Disc of head and pronotum without punctuation or pubescence, their surfaces with microsculpture of transverse waves and faint micropunctuation. Elytra with sparse punctuation and brown to black pubescence, interspaces without distinct microsculpture. Abdomen moderately densely punctuated, without distinct metallic reflection, with brown to black pubescence.

Head wider than long, with tempora strongly tapered towards relatively narrow neck, about as long as eye (in lateral view) forming poorly distinct broadly rounded hind angles; neck not delimited from head dorsally. Antennae with antennomeres VIII–X distinctly wider than long, transverse.

Pronotum about as wide as long, and about as wide as head; pronotal anterior and posterior angles rounded but distinct; pronotum widest in the area of its middle, its sides very gradually converging posteriad, and more strongly converging anteriad. Pronotal hypomera inflexed, but slightly visible from lateral view.

Elytron elongate, longer than pronotum.

Wings well developed.

Abdominal tergites III–V (first to third visible) with moderately deep transverse impression in basal part; tergite VII (fifth visible) with whitish seam at apical margin.

Male unknown.

#### Comparison.

Antimerus gracilis is most similar to Antimerus jamesrodmani, from which it differs in coloration (cf. Figs 18 and 16) and in the slightly more pronounced posterior angles of the head. From Antimerus bellus, another similar species, Antimerus gracilis also differs in coloration of the body (cf. Figs 18 and 17).

#### Distribution and bionomics.

Antimerus gracilis is known only from the type locality in northeastern Queensland (Fig. 55, B, square). The only available specimen was collected by low-scale fogging of trees from the ground, in the forest at the elevation 1325 m.

#### Etymology.

The name of the new species is from the Latin adjective *gracilis*, or slender.

### Antimerus monteithi sp. n.

urn:lsid:zoobank.org:act:34CE4E42-068F-4827-ADE6-EB71196D11D9

Figs 15 40–42 

#### Type locality:

Australia: Queensland: Springbrook Repeater, 1000m, 28°14’S, 153°16’E

#### Material examined.

**AUSTRALIA: Queensland:** Holotype, pinned, genitalia not dissected but aedeagus protruding from abdomen, with labels: “SEQ: 28°14’S x 153°16’E / Springbrook Repeater,/ 3 Oct-31 Dec 1997/. G. Monteith 1000m/ Rainfor. Intercept 5648”, “QUEENSLAND/ MUSEUM LOAN/ DATE: Sept. 2004/ No. LE 04.51”, “FMNH-INS/ 0000 019 175”, “Antimerus/ n.sp. 3/ det. A. Newton 2004”/ “HOLOTYPE/ Antimerus/ monteithi sp.n./ A. Solodovnikov des. 2006”, 1♂ in QM. Paratypes:Lamington N.P., Binna Burra, 18.xi.1982, on logs in rainforest (S. Endrödy-Younga), FMNH-INS 0000 019 999, 1♂ in TMSA; Lamington N.P., O’Reillys, Border Trail, 920–1000 m, 960m, 11.i.1991, under bark (Pollock & Reichardt), FMNH-INS 0000 019 142, 1♀ in FMNH; **New South Wales:** Acacia Plateau & Wilson’s Peak area - Koreelah St. For., >900m, 28°16’S, 152°27’E, dry subtropical rainforest, exposed slope, 11.xii.1988, pitfall trap AP3 (Smith, Hines, Pugh & Webber), 1♂ in AMS.

#### Description.

Measurements (n=3): HL: 1.8–2.6; HW: 2.5–3.0; PL: 2.3–2.7; PW: 2.8–3.3; EL: 3.0–3.3; EW: 2.9–3.3. Total length of the body: 13–15 mm.

Head and pronotum black, glossy but without metallic reflection; elytra blue-greenish, glossy, with strong metallic reflection; abdomen and appendages dark brown to black. Disc of head and pronotum without punctuation or pubescence, their surfaces with microsculpture of transverse waves and faint micropunctuation. Elytra with irregular, non-setiferous punctuation, glabrous with only a few long black macrosetae; interspaces between punctures without distinct microsculpture. Abdomen densely and moderately coarsely punctate, without metallic reflection, with brown to black pubescence; interspaces between punctures with distinct transverse microsculpture.

Head wider than long; tempora about as long as eye (in lateral view) forming distinct broadly rounded hind angles. Antennae with antennomeres VIII–X distinctly wider than long, transverse.

Pronotum distinctly transverse, obviously wider than long and about as wide as head; pronotal posterior and especially anterior angles very distinct; pronotum widest in the area near its posterior angles, gradually narrowing anteriad. Pronotal hypomera strongly inflexed, not visible from lateral view.

Elytron longer than pronotum, about as long as wide.

Wings well developed.

Abdominal tergites III–V (first to third visible) with slight transverse impression in basal part; tergite VII (fifth visible) with whitish seam at apical margin.

**Figures 40–42.**
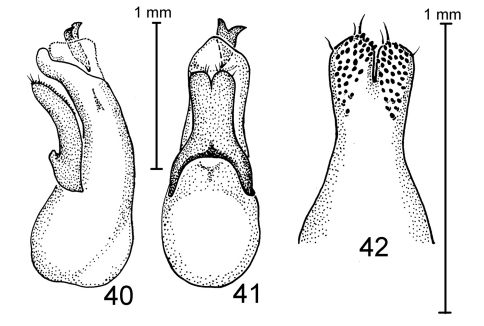
Antimerus monteithi, aedeagus: **40** aedeagus laterally **41** aedeagus dorsally (parameral side) **42** apical portion of paramere, underside.

Male (Figs 40–42). Aedeagus with paramere slightly narrower than median lobe (in dorsal view), distinctly bilobed; lobes separated by deep narrow incision.

#### Comparison.

Antimerus monteithi is a very distinct species, that can be easily distinguished from any other member of the genus by having completely glabrous elytra and a distinctly transverse pronotum with very distinct anterior angles, giving it a quediine-like appearance.

#### Distribution and bionomics.

Antimerus monteithi is known from four neighboring localities in the border area between southeastern Queensland and northeastern New South Wales (Fig. 55, C, squares). All specimens were taken in the forest at an elevation of 900–1000 m. Two specimens were collected on logs or under tree bark, one by pitfall trap and one at a flight intercept trap.

#### Etymology.

We dedicate this species to our friend and colleague Geoff Monteith to acknowledge his great collecting effort in Australia, which has made significant material on Staphylinidae from this continent available for study. In particular, he collected the holotype of this peculiar species.

## Larvae of Antimerus

The life cycle of Antimerus species is unknown, and no immature stages have been described. A very distinctive larval type of Staphylininae of large size but unknown identity has been found in numerous forest litter samples in eastern Australia, from northern Queensland to southern Victoria (Fig. 55), within the general range of various Antimerus species but not in direct association with adults. We attribute these larvae to Antimerus for the following reasons: 1) The larvae appear to fall into three size classes, probably representing the usual three instars of known larvae of Staphylininae. The largest larvae, presumably of the final instar, are about 16 mm long with heads about 3 mm wide. There are few adult Staphylininae in eastern Australia large enough to belong to these larvae, and most of these belong to genera for which some larvae are known (Creophilus Leach, 1819; Hesperus Fauvel, 1874; Quedius Stephens, 1829*, Tasgius* Stephens, 1829and Thyreocephalus Guérin-Méneville, 1844), or in which known species are restricted to northern Queensland (Actinus Fauvel, 1878 and Mysolius Fauvel, 1878), leaving only Antimerus species, Lonia regalis (Olliff, 1887) and Australotarsius grandis Solodovnikov & Newton, 2009 as possibilities based on size and distribution. 2) The larvae clearly belong to Staphylininae and probably Staphylinini (see description below), but, like Antimerus adults, do not fit any of the recognized subtribal groups very well. 3) The larvae have been found at many of the same localities at which one or more species of adults of Antimerus have been found, and all were found within the known ranges of various Antimerus species. In contrast, adults of Lonia regalis are confirmed as present only in southern Queensland and northern New South Wales (Fig. 55, D), and are exceedingly rare; to our knowledge, adults have not been collected by anyone since the 1920’s. Australotarsius grandis is also known only from a few specimens originating in a few localities in Queensland and New South Wales (Solodovnikov and Newton 2009). 4) The larvae apparently represent at least three species, whose distributions correlate approximately with the known distributions of one or more Antimerus species; Lonia is a monobasic genus, and Australotarsius grandis is the only species in that genus which nears the size of Antimerus. For these reasons, the larvae described below are attributed to Antimerus.

These larvae are very similar but not identical. Based on their distributions and characteristics, they apparently represent three species (see provisional key below). The Victoria larvae are referred to Antimerus smaragdinus, the only species known from Victoria. The Kirrama S.F. larvae are referred to Antimerus metallicus because adults have been collected at the same locality and this is the only common species in northern Queensland. The rest are referred to Antimerus punctipennis, the commonest species by far throughout New South Wales and southern Queensland; adults of this species have often been found at the same localities as these larvae. Adults of these three Antimerus species are closely similar and replace one another geographically, so it would not be surprising to find that their larvae are also very similar.

### Diagnostic description

Large Staphylininae (length ca. 8–16 mm, head width 1.5–3.0 mm), with large subquadrate well-sclerotized head, much narrower thorax, and fusiform abdomen which at middle may exceed head width in well-fed larvae (Fig. 19). Body surfaces and appendages generally microspinose or microtuberculate and with sparse to fairly dense fine simple setae; most macrosetae and many intermediate-sized setae club-shaped with “frayed” or multispinose apices.

**Figures 43–54.**
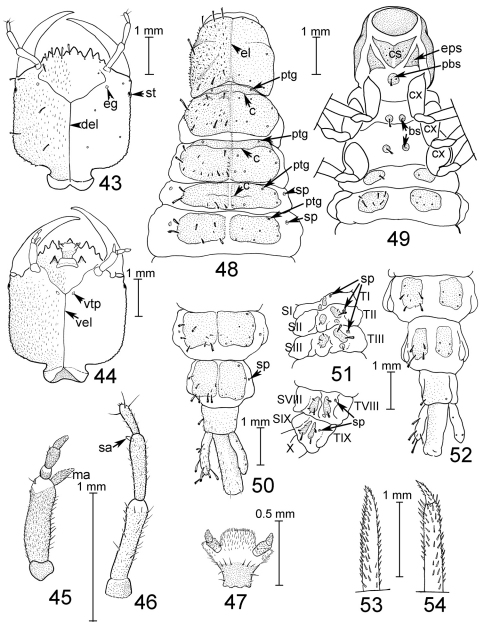
Antimerus punctipennis, presumed larva (instar III): **43** head dorsally **44** head ventrally **45** right maxilla ventrally **46** right antenna dorsally **47** labium ventrally o abdominal segments, ventrally **50** abdominal segments VII–X, dorsally **51** abdominal segments I–III and VIII–IX, laterally **52** abdominal segments VII–X, ventrally **53** anterior leg, apical part **54** middle leg, apical part; *bs,* basisternites; *c,* carina; *cs,* cervicosternum; *cx,* coxae; *del,* dorsal ecdysial lines; *el,* ecdysial line; *eg,* epicranial gland; *eps,* proepisternum; *ma,* mala; *pbs,* probasisternum; *ptg,* protergal gland; *sa,* main sensory appendage; *sp,* spiracle*; st,* stemma; *SI–IX,* abdominal sternites I–IX; *TI–IX,* abdominal tergites I–IX; *vel,* ventral ecdysial lines; *vtp,* ventral tentorial pits.

Head (Figs 43, 44) subquadrate, well sclerotized, light to dark brown, with a single large pigmented stemma (Fig. 43, st) on each side posterior to dorsal mandibular articulation and separated from that by less than basal width of mandible (in better-preserved larvae the pigment is apparently formed by near-fusion of 3–4 pigment spots); neck distinct and narrow, about 1/3 as wide as head; dorsal ecdysial lines lyriform, ending near antennal foramen (Fig. 43, del); epicranial gland (Fig. 43, eg) present; nasale with 9 acute teeth of different sizes; epipharynx sclerotized, without membranous setose areas except adjacent to pharynx; antenna (Figs 43, 46) about half as long as head width, 4-segmented, basal antennomere transverse, others elongate, main sensory appendage of antennomere 3 (Fig. 46, sa) ventral; mandible long, falcate, edentate; maxilla with 3-segmented palp and elongate mala (longer than any palpal segment) (Fig. 45, ma); labium (Fig. 47) with transverse ventral premental sclerite, 2-segmented palps, and unsclerotized microspinose trilobed apex including transverse median ligula and acute lateral lobes on which the palps are inserted; ventral ecdysial lines Y-shaped, split at level of ventral tentorial pits and ending near maxillary foramen (Fig. 44, vel).

Pronotum about 2/3 as wide as head, meso- and metanotum wider but not as wide as head, abdomen wider still (at about segment III–IV may exceed head width) (Fig. 48) then gradually narrowed to apex. Meso- and metanotum each with subbasal transverse carina (Fig. 48, c). Thoracic terga with mid-longitudinal ecdysial line (Fig. 48, el), abdominal terga and sterna I–VIII more widely divided along midline. Abdominal segments II–VIII each with two pairs of lateral sclerites (dorsal and ventral) (Fig. 51). Urogomphus robust, apparently 1-segmented (except Antimerus smaragdinus with a weakly articulated small apical segment), about 2/3 as long as segment X which is at least 3X longer than wide and bears eversible spinose membranous lobes at apex (Figs 50, 52). Cervicosternum large, triangular, vaguely divided along midline (Fig. 49, cs); probasisternum a single sclerite (vaguely divided along midline in some specimens) (Figs 49, pbs), meso- and metathorax each with a pair of small basisternites (Fig. 49, bs); proepisternum large, triangular, subequal in size to half of cervicosternum (Fig. 49, eps); mes- and metepisterna each smaller, transverse. Front leg with tarsungulus fused indistinguishably to tibia (Fig. 53), meso- and metalegs each with free, multisetose tarsungulus (Fig. 54); pro- and mesotibiae of all instars with multiple short specialized setae bearing several spines each on anterior face.

### Provisional key to Antimerus species (larvae)

**Table:** 

1.	Posterolateral macrosetae of abdominal tergites slender, weakly club-shaped, the setae subequal in length to length of tergite, the apex of each seta less than twice as wide as base of seta; urogomphus with more or less distinct small apical segment [instars I and II only seen] (southeastern Australia, Tasmania)	Antimerus smaragdinus
–	Posterolateral macrosetae of abdominal tergites stout, strongly club-shaped, the setae shorter than length of tergite, the apex of each seta more than twice as wide as base of seta; urogomphus apparently 1-segmented	2
2(1).	Macrosetae very short and stout, posterolateral macrosetae of abdominal tergites less than 1/3 as long as tergite in instar III (southern Queensland, New South Wales)	Antimerus punctipennis
–	Macrosetae more elongate, posterolateral macrosetae of abdominal tergites more than half as long as tergite in instar III [instar III only seen] (northern Queensland)	Antimerus metallicus

### Larval material examined

(L-I, L-II, L-III indicates estimated larval instar I, instar II and instar III, respectively)

Antimerus punctipennis **Lea?: AUSTRALIA: New South Wales:** Barrington Tops, Mt. Allyn-Burraga Tr., 1000m, 16.vii.1978, Nothofagus litter (S. & J. Peck), 1L-III in FMNH; Dorrigo N.P., E end Blackbutt Tr., Never Never Picnic Area, 710m, ANMT 589, 30°22’S, 152°48’E, subtropical rainforest, 28.ii.1980, FMHD#80-333, berl., litter (A. Newton & M. Thayer), 1L-I in FMNH; 28.ii.1980, FMHD#80-332, berl., rotting fruits Endiandra introsa (A. Newton & M. Thayer), 1L-I in FMNH; Mt. Keira scout camp, 320m, rainforest, 4–5.iii.1981, ANIC 707–708, Berlesate leaf/log litter (Lawrence & Calder), 1L-I, 1L-II in FMNH; Royal N.P., 34°7’S, 151°3’E, rainforest, 5.vi.1978, berl., leaf litter with hyphae (S. & J. Peck), 1L-II, 1L-III in FMNH; Wiangaree S.F. [now Border Ranges N.P.], Brindle Creek, 740m, ANMT 592, 28°23’S, 153°3’E, subtropical rainforest w/Nothofagus, Araucaria, 29.ii–3.iii.1980, FMHD#80-340, berl., litter (A. Newton & M. Thayer), 6L-I in FMNH; Wiangaree S.F. [now Border Ranges N.P.], Isaksson Ridge, 1050m, ANMT 593, 28°22’S, 153°6’E, Nothofagus moorei rainforest, 2.iii.1980, FMHD#80-339, berl., litter (A. Newton & M. Thayer), 3L-I in FMNH; Wiangaree S.F. [now Border Ranges N.P.], Sheepstation Creek, 600m, ANMT 591, 28°24’S, 153°2’E, subtropical rainforest, 29.ii.1980, FMHD#80-334, berl., litter (A. Newton & M. Thayer), 1L-I in FMNH; **Queensland:** Joalah N.P., rainforest, 20.x.1978, ANIC 653, berl., litter (Lawrence & Weir), 1L-III in ANIC, 1L-III in FMNH; Antimerus smaragdinus **Fauvel?: AUSTRALIA: Victoria:** Wilson’s Promontory N.P., Lilly Pilly Trail, 3m, 39°1’S, 146°19’E, 13.v.1978, FMHD#78-152, Eugenia litter (S. & J. Peck), 1L-I, 1L-II in FMNH; Antimerus metallicus **sp. n.?: AUSTRALIA: Queensland:** Kirrama S.F., Kirrama Range Rd., Bridge 10 vic., 690–720 m, ANMT 1137, 18°12.88’S, 145°48.38’E, rainforest near stream, 4.ix.2004, FMHD#2004-208, berl., litter (D. Clarke, A. Newton & M. Thayer), 1L-III in FMNH; Kirrama S.F., Society Flat Rainforest Walk, 580m, ANMT 1136, 18°12.167’S, 145°44.981’E, rainforest with Euc. grandis & Agathis emergents, 4.ix.2004, FMHD#2004-207, berl., bark, log, & leaf litter (M. Thayer, A. Newton & D. Clarke), 1L-III in FMNH.

**Figure 55.**
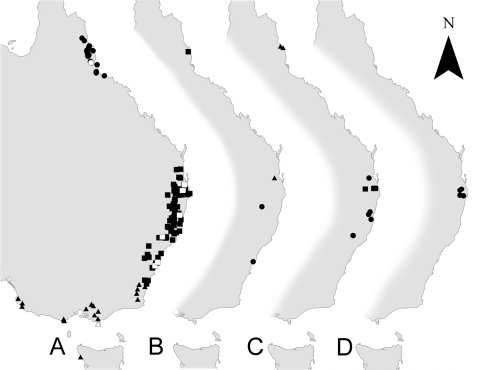
Distribution of Antimerus (A, B, C) and Lonia (D), black symbols – adults, white symbols – larvae: **A** Antimerus punctipennis group: Antimerus metallicus (circles), Antimerus punctipennis (squares) and Antimerus smaragdinus (triangles) **B** Antimerus jamesrodmani group: Antimerus gracilis (square), Antimerus jamesrodmani (triangle) and Antimerus bellus (circles) **C** Antimerus auricomus group: Antimerus posttibialis (triangles) and Antimerus auricomus (circles), and Antimerus monteithi group: Antimerus monteithi (squares) **D** Lonia regalis (circles).

### Comments on larval morphology of Antimerus

Larvae have the general characteristics of Staphylininae, including the unique, large, triangular cervicosternum, and the general characteristics of Staphylinini, including the very elongate pygopod (abdominal segment X), presence of many specialized “frayed” setae on the body, and indications of the derivation of the single stemma from fusion of four stemmata. However, they have several unique features within this subfamily and tribe, including an unsclerotized ligula (reminiscent of Paederinae, and especially the broad ligula of Pinophilus and trilobed ligula of Hyperomma), and the unique feature of fusion of the tarsungulus and tibia of the front leg. Other features of both adults and larvae of Antimerus species are reminiscent of the genus Pinophilus and related genera of Paederinae: Long falcate mandibles, short labrum, short wide head and enlarged “sticky” tarsi of adults, and large quadrangular head, small body and broad ligula of larvae.

**Figure 56.**
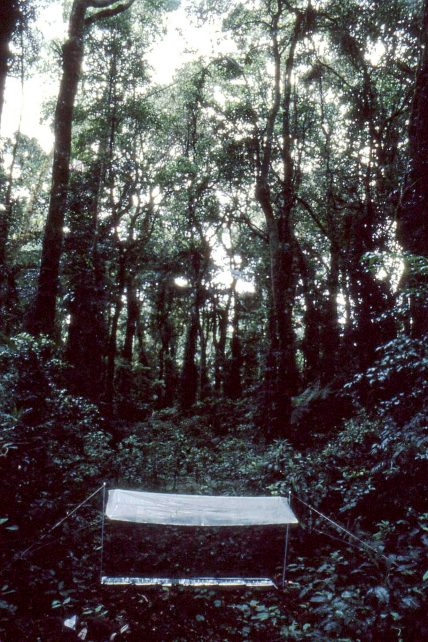
Habitat of Antimerus. Flight intercept trap in warm temperate rainforest at 1000 m elevation in Border Ranges National Park (near junction of Tweed Range and Brindle Creek Roads), northern New South Wales. Four males of Antimerus punctipennis were collected in this trap and one female found in adjacent leaf litter, in early January. Ten first-instar larvae attributed to this species were found at three nearby sites in the park, in leaf litter of similar or subtropical rainforest, at 600–1050 m elevation, in late February–early March (photo: A.F. Newton).

## Discussion

Revision of all available material of the genus Antimerus, including substantial collections made in recent years, more than doubled the number of species hitherto known for the genus. Still, most of the species are known only from a very limited number of specimens. A presumably arboreal way of life of at least some species of Antimerus, which would make these beetles unlikely to be collected by the most commonly used collecting techniques, allows us to expect the discovery of additional new species, especially among material collected from the forest canopy.

The high degree of morphological diversity displayed within the genus Antimerus is also noteworthy. Based on their morphology (especially with reference to the characters used in the key to adults, above), the species of the genus can be arranged into preliminary species groups as follows (groups named after the first-listed species each): 1) *auricomus* + *posttibialis*; 2) *smaragdinus* + *punctipennis* + *metallicus*; 3) *jamesrodmani* + *bellus* + *gracilis*; and 4) *monteithi*. Within each species group, species have a similar morphology and allopatric distributions. Species from different species-groups, on the contrary, are more remote morphologically and the distributions of the entire species-groups are largely overlapping. These patterns apparently indicate a long divergence time and dispersal history of these respective lineages within the genus, both lines of evidence pointing to a long existence of the genus as a whole. However, neither the pattern of morphological divergence within this peculiar genus, nor its distribution patterns, can give a precise enough measure of the age of the genus to shed light on the phylogenetic problem outlined in the Introduction (i.e., whether Antimerus is a very basal lineage of Staphylinini, or a younger genus, basal within the “Staphylinini propria” clade.). The fact that Antimerus is known to be distributed only in the humid forests of eastern Australia, and missing from those of the southwestern corner of that continent, argues against the possibility of an older, Gondwanan, age for this genus. (Several genera of the presumably much older, Gondwanan “Austral Quediina” group occur both in the eastern and southwestern parts of Australia). Such a distribution of Antimerus in eastern Australia only is more consistent with the latter assumption, that Antimerus is a basal member of the “Staphylinini propria” clade. As pointed out in the introduction, diverse data suggest that the “Staphylinini propria” clade within Staphylinini originated in the northern hemisphere landmasses in times when those were already separated from Gondwana. Assuming that Antimerus is a member of “Staphylinini propria”, it may have colonized Australia from southeast Asia, where subsequently it apparently went extinct. This scenario suggests that additional species may yet be discovered in southeast Asia or New Guinea.

The newly discovered putative larvae of Antimerus have a very peculiar morphology, suggesting this genus may be even more isolated within Staphylinini than adult morphology suggests. Larval morphology alone is more consistent with the idea that Antimerus is a more ancient lineage, not belonging to the “Staphylinini propria” clade. But too many larval forms of Staphylinini are still unknown to allow any sound conclusions at present. In addition, we interpret many larval features of Antimerus, especially some similarities with larvae of pinophiline Paederinae, as a result of convergent specialization, possibly due to a similar lifestyle and/or prey specialization. Unfortunately, beyond the association of at least some species of Antimerus and Pinophilini adults with foliage, these lifestyles and specializations remain unknown in both groups.

## Supplementary Material

XML Treatment for Antimerus

XML Treatment for Antimerus auricomus

XML Treatment for Antimerus posttibialis

XML Treatment for Antimerus smaragdinus

XML Treatment for Antimerus punctipennis

XML Treatment for Antimerus metallicus

XML Treatment for Antimerus jamesrodmani

XML Treatment for Antimerus bellus

XML Treatment for Antimerus gracilis

XML Treatment for Antimerus monteithi
